# Unraveling Novel Mechanisms of Neurodegeneration Through a Large-Scale Forward Genetic Screen in *Drosophila*

**DOI:** 10.3389/fgene.2018.00700

**Published:** 2019-01-14

**Authors:** Samantha L. Deal, Shinya Yamamoto

**Affiliations:** ^1^Program in Developmental Biology, Baylor College of Medicine, Houston, TX, United States; ^2^Department of Molecular and Human Genetics, Baylor College of Medicine, Houston, TX, United States; ^3^Department of Neuroscience, Baylor College of Medicine, Houston, TX, United States; ^4^Jan and Dan Duncan Neurological Research Institute, Texas Children’s Hospital, Houston, TX, United States

**Keywords:** neurodegeneration, *Drosophila melanogaster*, Mendelian diseases, mitochondria, reactive oxygen species, iron accumulation, endolysosomal trafficking, autophagy

## Abstract

Neurodegeneration is characterized by progressive loss of neurons. Genetic and environmental factors both contribute to demise of neurons, leading to diverse devastating cognitive and motor disorders, including Alzheimer’s and Parkinson’s diseases in humans. Over the past few decades, the fruit fly, *Drosophila melanogaster*, has become an integral tool to understand the molecular, cellular and genetic mechanisms underlying neurodegeneration. Extensive tools and sophisticated technologies allow *Drosophila* geneticists to identify and study evolutionarily conserved genes that are essential for neural maintenance. In this review, we will focus on a large-scale mosaic forward genetic screen on the fly X-chromosome that led to the identification of a number of essential genes that exhibit neurodegenerative phenotypes when mutated. Most genes identified from this screen are evolutionarily conserved and many have been linked to human diseases with neurological presentations. Systematic electrophysiological and ultrastructural characterization of mutant tissue in the context of the *Drosophila* visual system, followed by a series of experiments to understand the mechanism of neurodegeneration in each mutant led to the discovery of novel molecular pathways that are required for neuronal integrity. Defects in mitochondrial function, lipid and iron metabolism, protein trafficking and autophagy are recurrent themes, suggesting that insults that eventually lead to neurodegeneration may converge on a set of evolutionarily conserved cellular processes. Insights from these studies have contributed to our understanding of known neurodegenerative diseases such as Leigh syndrome and Friedreich’s ataxia and have also led to the identification of new human diseases. By discovering new genes required for neural maintenance in flies and working with clinicians to identify patients with deleterious variants in the orthologous human genes, *Drosophila* biologists can play an active role in personalized medicine.

## Contributions of *Drosophila* to Neurodegeneration Research

Neurodegenerative diseases are characterized by the progressive loss of neurons, leading to the deterioration of motor and/or cognitive functions. Since one of the largest risk factors for neurodegenerative disorders is age (Niccoli and Partridge, 2012), the prevalence of these diseases is increasing in many countries. Although underappreciated, Alzheimer’s disease (AD) is the sixth leading cause of death in the United States (James et al., 2014; Weuve et al., 2014). In addition, many people are impacted by degenerative motor disorders such as Parkinson’s disease (PD), amyotrophic lateral sclerosis (ALS), spinocerebellar ataxias (SCA), Huntington’s disease (HD), and multiple sclerosis (MS). Life-threatening neurodegenerative diseases are also seen in neonatal and pediatric clinics, including Leigh syndrome, Zellweger syndrome, Friedreich’s ataxia, spinal muscular atrophy and lysosomal storage disorders. Hence, neurodegenerative diseases affect not only the ever-increasing aging population, but also a number of infants and children worldwide.

Genetic causes of many neurodegenerative diseases have been identified through pedigree analysis and more recently through next-generation sequencing efforts (Chong et al., 2015; Boycott et al., 2017). By studying the function of these genes and assessing the impact of disease-associated variants, researchers are working to understand the molecular causes of neurodegenerative disorders. In addition to experiments performed in human cells and in mice, functional studies of neurodegenerative disease-associated genes in the fruit fly *Drosophila melanogaster* have provided insights into molecular mechanisms of neuronal maintenance and deterioration (Şentürk and Bellen, 2018). There are two complementary strategies to tackle neurodegenerative diseases using flies. The first strategy uses *Drosophila* to over-express pathogenic factors in the nervous system to recapitulate some of the cellular and histological features seen in the human condition. Over-expression of pathogenic proteins, peptides or RNAs can cause the degeneration of fly neurons. This is often accompanied by shortened life span and a number of behavioral phenotypes such as defects in climbing, flight, learning, and memory. This approach has been used to study Amyloid Precursor Protein (AD) (Greeve et al., 2004), Aβ42 (AD) (Zhao et al., 2010), α-Synuclein (PD) (Chen and Feany, 2005; Roy and Jackson, 2014), Huntingtin (HD) (Jimenez-Sanchez et al., 2015), Tau [Frontotemporal Dementia (FTD)] (Frost et al., 2014), Superoxide Dismutase 1 (ALS) (acp:c8Sahin et al.Şahin et al., 2017), Ataxin-1 (SCA1) (Shiraishi et al., 2014), and C9orf72 (ALS/FTD) (Mizielinska et al., 2014) in the fly nervous system using the GAL4/UAS binary expression system (Brand and Perrimon, 1993). Over-expression of these factors in the developing visual system often produces a ‘rough eye’ phenotype, providing researchers with an easily scorable assay for high-throughput screens (Lenz et al., 2013). This strategy has been effective for studying diseases caused by gain-of-function mechanisms, and it provides a platform to identify genes and genetic pathways that function as genetic suppressors or enhancers. Such screens have provided putative drug targets (Park et al., 2013; Jimenez-Sanchez et al., 2015; Marcogliese et al., 2017) and have the potential to identify genetic modifiers that influence the penetrance or expressivity of disease phenotypes in humans.

The second strategy uses *Drosophila* to identify genes that are necessary for maintenance of the fly nervous system through loss-of-function (LOF) approaches. This methodology was introduced in the 1970s through forward genetic screens in Seymour Benzer’s laboratory (Anderson and Brenner, 2008). In a pioneering study, Hotta and Benzer (1972) used a chemical mutagen ethyl methanesulfonate (EMS) to induce random mutations in the fly genome, and screened for mutants that developed normally but exhibited age-dependent defects. Subsequent pathological studies of the brain revealed that behavioral defects were associated with histological signs of neurodegeneration such as vacuolization. This screen led to identification of genes like *drop dead* (Buchanan and Benzer, 1993) and *swiss cheese* (Kretzschmar et al., 1997), named for their short life span and brain pathology phenotypes, respectively. Although not all genes identified through these screens are evolutionarily conserved, some have clear human orthologs that are linked to neurodegenerative disorders. For example, the *swiss cheese* ortholog *PNPLA6* (*Patatin-Like Phospholipase Domain-Containing Protein 6*) is linked to a number of human diseases that exhibit neurological symptoms including Boucher-Neuhauser syndrome (OMIM #215470), Laurence-Moon syndrome (OMIM #245800), Oliver-McFarlane syndrome (OMIM #275400) and autosomal recessive spastic paraplegia type 39 (OMIM #612020). The molecular cloning of the *swiss cheese* gene in *Drosophila* (Kretzschmar et al., 1997) and subsequent identification of the human ortholog (Lush et al., 1998) laid the groundwork to understand the molecular pathogenesis of these disorders (Rainier et al., 2008). Moreover, PNPLA6 is a critical mediator of organophosphate poisoning leading to neurodegeneration (O’Callaghan, 2003; Winrow et al., 2003). Hence, *Drosophila* research not only facilitates the study of rare genetic disorders but can also unravel mechanisms underlying neurodegenerative disorders caused by environmental factors including pesticides and warfare agents.

While studies identifying fly mutants with neurodegenerative phenotypes have led to important discoveries, these screens are very time consuming. Moreover, it is often challenging to identify the causative molecular lesion responsible for the neurodegenerative phenotype since mutagens such as EMS introduce a large number of mutations throughout the genome (Blumenstiel et al., 2009; Gonzalez et al., 2012). Even with high-throughput sequencing technologies, one needs to genetically map the trait of interest to a small chromosomal region to interpret the sequencing results (Haelterman et al., 2014a). Considering that genetic mapping can be quite labor intensive for age-dependent phenotypes, this methodology is not easily scalable. Furthermore, these types of screens will miss genes that are required for development as well as neuronal maintenance due to early lethality. The post-developmental function of genes that are essential for development and survival (essential genes) can be studied through tissue specific gene knockdown approaches using RNA interference (RNAi). For example, complete loss of *Presenilin*, the fly ortholog of *PSEN1* and *PSEN2* that are associated with AD, causes larval lethality due to the gene’s role in Notch signaling (Guo et al., 1999). However, neuronal knockdown of *Presenilin* or other γ-secretase complex subunits such as *Nicastrin* revealed that γ-secretase activity is essential for neuronal maintenance. These flies exhibited shortened life-span and age-dependent climbing defects accompanied by histological signs of neurodegeneration (Kang et al., 2017). Interestingly, brain specific knockout of *Psen1*, *Psen2* or *Nicastrin* in mice also results in neurodegenerative features independent of β-amyloid accumulation (Beglopoulos et al., 2004; Feng et al., 2004; Saura et al., 2004; Tabuchi et al., 2009), pointing to an evolutionarily conserved function of the γ-secretase complex in neuronal maintenance. Hence, uncovering neuroprotective functions of essential genes can provide insights into molecular mechanisms of neurodegeneration across species.

**FIGURE 1:**
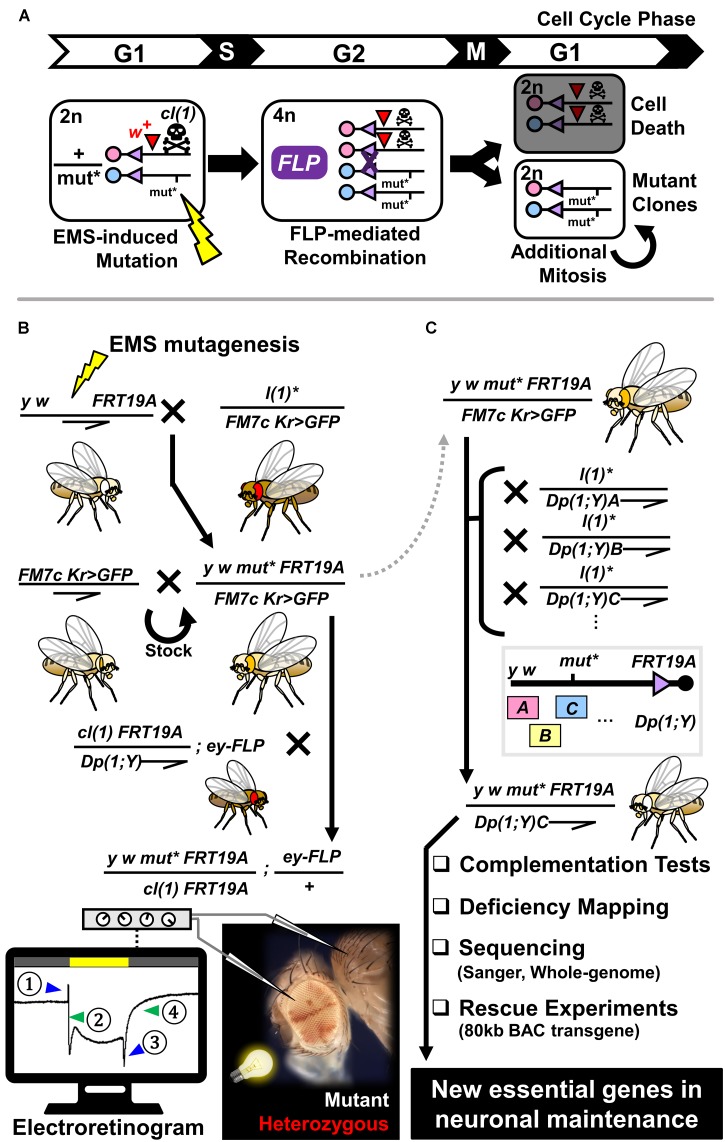
Generation of mosaic flies using the FLP/FRT-mediated mitotic recombination system and flowchart of an adult mosaic screen to identify mutants that exhibit neurodegenerative phenotypes using electroretinogram. **(A)** Schematic diagram of generation of a homozygous mutant clone from a cell that is heterozygous for a mutation of interest (*mut^∗^*) induced by random mutagenesis via EMS. During the G2 phase of the cell cycle, tissue specific expression of FLP can mediate recombination between two sister chromosome arms that contain FRT sites. ∼50% of the time, one of the daughter cells will become homozygous for the mutation of interest, whereas the sibling cell is homozygous wild-type for the same gene. By including a recessive cell lethal mutation [*cl(1)*] on the opposite chromosome strand, one can eliminate the homozygous wild-type cell to give a growth advantage to the mutant cell. A dominant eye color marker (*w^+^*) allows labeling of the heterozygous clones with red eye pigments. **(B)** Crossing scheme to generate stable stocks that carry X-linked mutations on an FRT chromosome and further test their impact on neuronal maintenance in the fly visual system. Through a three-generation cross, we obtained flies that are mosaic for recessive lethal mutations on the X-chromosome. By performing ERG on young (∼3 day post-eclosion) and aged (∼3 week post-eclosion) animals, we determined whether these flies show age-dependent decline in neuronal function. Schematic diagram of ERG recording from a fly with mosaic eyes is shown at the bottom. By placing a field recording electrode on the mutant region (white patches), inserting a reference electrode in the thorax, and shining a white light for 1 s, we record the activity of photoreceptors and post-synaptic neurons. ERG traces can be subdivided into four components; (1) on-transient, (2) depolarization, (3) off-transient, and (4) repolarization. Alterations in (2) and (4) are indicative of defects in phototransduction or neuronal health, whereas defects in (1) and (3) reflect issues in synaptic wiring or communication between the eye and the brain. **(C)** Strategy to map recessive lethal mutations that are linked to neurodegenerative phenotypes. Mutations of interest are maintained as a stable stock using a balancer chromosome (*FM7c Kr > GFP*). By crossing heterozygous female flies to male flies that carry a series of X-chromosome duplications that are translocated on the Y chromosome or autosomes, we attempted to identify a duplication that can rescue the lethality of hemizygous mutant males. Once such duplication is identified, we used the rescued males to perform complementation tests and deficiency mapping in order to further map the mutation to a small region on the X-chromosome. By sequencing exons or whole-genomes of mutant flies and performing rescue experiments using BAC transgenic flies, we mapped hundreds of mutations to single genes through this approach.

An alternative strategy to study essential genes in neuronal maintenance is by generating flies that are mosaic for a mutation of interest. Analogous to the Cre/loxP system used in the mouse field (Gu et al., 1993), *Drosophila* researchers utilize the FLP/FRT (FLiPpase/FLP Recombinant Target)-mediated recombination system to generate mosaic animals (Golic and Lindquist, 1989). In contrast to the Cre/loxP system, which uses two loxP sites *in cis* flanking a critical exon to generate conditional knockout cells in tissues that express the Cre recombinase, the FLP/FRT system uses two FRT sites *in trans* that are located in the identical locus on two sister chromosomes. One chromosome contains a mutation of interest, and the other chromosome contains a scorable marker [e.g., *white^+^* (*w^+^*), *yellow^+^* (*y^+^*), GFP]. Upon expression of the FLP recombinase and subsequent cell division, homozygous mutant and wild-type daughter cells are generated from a heterozygous cell undergoing mitosis, a process known as mitotic recombination (Figure 1A). Moreover, by using a recessive cell lethal (cl) mutation on the opposite chromosome, one can eliminate the sibling cell that is wild-type for the gene of interest and give a growth advantage to the mutant cell in order to generate large mutant clones. The FLP/FRT system allows study of gene function in homozygous knockout (null) cells by generating mitotic clones of nonsense, frameshift or deletion mutations. In addition, it permits the study of missense mutations, which may have different functional consequences from the null allele.

Developmental biologists in the late 1990s pioneered forward genetic screens based on FLP/FRT technology (St Johnston, 2002). Mosaic screens were initially used to isolate mutants affecting fundamental developmental processes like oogenesis (Duffy et al., 1998), embryonic patterning (Luschnig et al., 2004), integrin-mediated cell adhesion (Walsh and Brown, 1998), and signaling pathways (Végh and Basler, 2003; Jafar-Nejad et al., 2005). Further development of MARCM (Mosaic Analysis with a Repressible Cell Marker) technology permitted researchers to positively label mutant clones with fluorescent markers, allowing more detailed characterization of mutant phenotypes especially in the nervous system (Lee et al., 2000). Over the past 20 years, mosaic screens have been used to study neuronal phenotypes such as defects in neural stem cell division (Slack et al., 2006), synaptic transmission (Stowers and Schwarz, 1999; Verstreken et al., 2003), neuronal morphogenesis (Reuter et al., 2003), and neuronal connectivity (Newsome et al., 2000; Berger et al., 2008). Such screens uncovered roles of essential *Drosophila* genes in diverse biological contexts and stimulated research of orthologous genes associated with rare human genetic diseases as well as common traits. In one example, Verstreken et al. identified alleles in a previously uncharacterized gene through a FLP/FRT screen on the 2L chromosome arm designed to isolate new genes involved in synaptic transmission (Verstreken et al., 2009). The authors named this gene *tweek* based on a cartoon character with characteristic shaking and twitching movements. They demonstrated that Tweek regulates phosphoinositide levels in synaptic terminals in order to maintain proper synaptic vesicle recycling. Recently, autosomal recessive mutations in the human *tweek* ortholog gene *KIAA1109* were found to cause Alkuraya-Kucinskas syndrome (MIM #617822) characterized by global developmental delay and severe neurological abnormalities (Alazami et al., 2015; Gueneau et al., 2018). In another example, similar FLP/FRT screens on the 2R chromosome arm from Ly et al. and Dickman et al. independently identified mutant alleles of a gene that encodes the accessory α2δ subunit of the *Drosophila* voltage-gated calcium channel (Dickman et al., 2008; Ly et al., 2008). One group named this gene *straightjacket* based on the immobile phenotype seen in mutant larvae (Ly et al., 2008), and initial studies from the two labs elucidated its role in proper localization and stabilization of the pore forming α1 subunit encoded by the *cacophony* gene. In an independent screen that aimed to identify genes involved in pain perception based on RNAi, Neely et al. found that neuronal knockdown of *straightjacket* decreases pain sensitivity in flies (Neely et al., 2010). They further showed that mice that lack a *stj* ortholog (*Cacna2d3*) exhibit impaired responses to acute heat pain and identified single nucleotide polymorphisms in *CACNA2D3* that are associated with pain sensitivity in humans. Although these and other studies highlight the value of mosaic forward genetic screens in *Drosophila*, only recently was this system adapted for studying essential genes that are critical for neuronal maintenance (Zhai et al., 2006; Gambis et al., 2011; Neukomm et al., 2014; Yamamoto et al., 2014).

In this review, we provide an overview of a large forward genetic screen that was performed in Hugo Bellen’s laboratory on the *Drosophila* X-chromosome to identify essential genes that cause neurodegenerative phenotypes when mutated in the fly visual system (Yamamoto et al., 2014). This screen lead to identification of hundreds of mutations that exhibited neurological and neurodegenerative phenotypes, and many were mapped to specific genes using a combination of classic genetic and state-of-the-art genomic technologies (Haelterman et al., 2014a). We discuss the benefit of performing a large-scale screen on a unified genetic background, a feature that allowed us to recognize similarities and differences between the diverse mutations recovered from a single screen, and we highlight several genes that were studied further for their mechanistic roles in neuronal maintenance and underscore their associations with human diseases. We refer the readers who are interested in additional neurodegeneration studies in *Drosophila* to the following review articles (Bilen and Bonini, 2005; Lessing and Bonini, 2009; Jaiswal et al., 2012; McGurk et al., 2015; Şentürk and Bellen, 2018; Xiong and Yu, 2018).

## A Large-Scale Mosaic Chemical Mutagenesis Screen on the *Drosophila* X-Chromosome to Identify Essential Genes That Are Required for Neuronal Maintenance

The *Drosophila* X-chromosome carries 2,178 protein-coding genes, which is ∼15% of the entire *Drosophila* genome^1^. Considering that ∼30% of fly genes are predicted to be essential (Nüsslein-Volhard, 1994; Miklos and Rubin, 1996), one expects about 650 genes on this chromosome to cause lethality when mutated. Based on information from FlyBase (Gramates et al., 2017), 290 X-linked genes have been associated with a lethal mutation to date (excluding lethality caused by only RNAi or an uncharacterized transposable element), suggesting that there are a significant number of essential genes on this chromosome yet to be identified and functionally characterized. It is important to note that there is no correlation between X-linked traits in *Drosophila* and in humans. Compared to autosomes, forward genetic studies for essential genes on the X-chromosome face unique challenges. One critical step in mapping randomly induced mutations to specific genes is to perform complementation tests. By crossing two mutant strains together and checking the lethality of transheterozygous flies, one can determine whether the two mutations are alleles of the same gene (transheterozygous lethal) or not (transheterozygous viable). Unfortunately, for the X-chromosome, this is not straightforward as hemizygous males are lethal and therefore cannot be used for crosses. Hence, while non-essential genes on the X-chromosome have been relatively well-studied, essential genes on the X-chromosome have been difficult to work with, especially in the context of chemical mutagen-mediated forward genetic screens.

To identify essential genes that are required for neuronal maintenance on the fly X-chromosome, we designed and performed a three generation (F3) screen using EMS-mutagenesis and FLP/FRT mediated mitotic recombination. The overall schematic of this screen, which we refer to as the ‘X-screen’ throughout this article, is depicted in Figure 1B. Prior to the screen, we isogenized FRT containing chromosomes with recessive eye and body color markers (*y w FRT19A*) to select a healthy strain on which we performed mutagenesis. This isogenization step assures that all mutations are induced on an identical genetic background. We performed mutagenesis by feeding male flies a low concentration of EMS (7.5–10 mM) to avoid induction of multiple lethal hits per chromosome. We established 31,530 stocks, each carrying unique mutations. 5,859 lines were recessive lethal (18.6% lethality). If essential genes on the X-chromosome are distributed randomly, we estimated that ∼88% of the recessive lethal lines from this screen carry a single lethal mutation per X-chromosome based on a Poisson distribution model (Wieschaus et al., 1984; Winkler et al., 2005). The fact that most mutant lines did not have second site lethal hits was important for subsequent mapping and phenotypic characterization.

To identify genes that are necessary for neuronal maintenance, we generated flies containing homozygous mutant clones for each recessive lethal mutation in the fly eye. We accomplished this using *ey(eyeless)-FLP*, a flippase that is expressed in eye-antenna imaginal discs (progenitor of the eye, antenna and majority of head structure) and parts of the brain that are relevant to the visual system (Newsome et al., 2000). To generate large mutant clones, we selectively killed the homozygous control cells using a recessive cell lethal mutation [labeled as *cl(1)* in Figures 1A,B] that also contained a visible marker [*white(w)^+^*]. Hence in mosaic animals, eye clones that are red (*w^+^*) represent cells that are transheterozygous for the mutant of interest and the *cl(1)*, which behave as control cells. Eye clones that are white (*w^-^*) represents cells that are homozygous for the mutant of interest. Unlike vertebrate photoreceptors that contain short axons that project locally within the retina, fly photoreceptors project long axons into the brain to connect directly to neurons in the lamina or the medulla (Melnattur and Lee, 2011). Hence, fly photoreceptors are considered to have properties of both photoreceptors and retinal ganglion cells (cells that relay the signal from the retina to the brain), and most researchers consider them as true neurons. To screen for neurodegenerative mutants, we aged the flies for 3 weeks after eclosion and recorded the electroretinograms (ERG) of homozygous mutant patches of cells (Figure 1B). ERG of the fly eye can be broken down into four major components: on-transient, depolarization, off-transient, and repolarization (Lauwers and Verstreken, 2018). Depolarization and repolarization reflect the activation and silencing of the phototransduction cascade, whereas on- and off-transients reflect post-synaptic responses from brain cells activated by the photoreceptor neurons (Wang and Montell, 2007). Abnormal traces of depolarization or repolarization suggest defects in phototransduction or overall integrity of the photoreceptor, whereas alterations in on- and off-transients suggest problems with synapse formation or function. When we observed abnormal ERGs in 3-week-old (aged) animals, we assessed whether similar defects were seen in 3-day-old (young) flies. If the mutation of interest affected photoreceptor development or function, we expected to observe a similar phenotype in both young and aged animals. However, when the mutation caused neurodegeneration, we observed a much stronger phenotype in aged animals compared to the young ones. From this screen, we identified ∼800 lines with ERG defects, ∼1/3 of which exhibited age-dependent phenotypes.

In parallel to this effort, and using the same collection of X-linked recessive lethal mutants, we assessed whether the same set of mutants tested for ERG defects may cause other phenotypes in developing tissues (Yamamoto et al., 2012, 2013; Charng et al., 2014; Cook et al., 2017). Patterning defects in the wing such as wing margin loss (notching) and vein defects are associated with developmental signaling pathways like Notch signaling, Wnt signaling, Hedgehog signaling and BMP/TGF-β signaling (Bier, 2005; Salazar and Yamamoto, 2018). Conversely, defects in bristle and eye morphogenesis are linked to critical regulators of neurogenesis (Charng et al., 2014). By examining the morphology of eye and head in flies with mutant clones induced by *ey-FLP*, and assessing the phenotype of homozygous clones in the fly wing and thorax using *Ubx(Ultrabithorax)-FLP* (Jafar-Nejad et al., 2005), we identified ∼1,500 lines with clear morphological defects. ∼300 of these lines exhibited both ERG and morphological phenotypes, and a significant number of mutants presented with more than one morphological defect, consistent with the idea that genes are often pleiotropic (Wangler et al., 2017). We retained ∼2,000 lines with interesting developmental or neuronal defects and subjected them to mapping. Phenotypic and mapping data of these lines can be downloaded from the FlyPush server at BCM^2^.

## Mapping of X-Screen Mutants by Combining Classic Genetics and Modern Genomics

Molecular mapping of EMS-induced mutations is a labor intensive and time-consuming process. EMS primarily causes mutations through guanine alkylation, resulting in G/C-to-A/T transition in the genomic DNA. However, due to low-fidelity repair mechanisms activated upon the massive DNA damage occurring during mutagen treatment, other types of mutations, such as transversions and indels, can also be introduced (Sega, 1984). Considering that most mutations are likely to be single base pair changes on the X-chromosome, we need to identify a needle (1 bp) in a large haystack (∼23,540,000 bp). Over the years, many laboratories have generated a number of tools to facilitate the mapping of mutations, some of which can be traced back to the beginning of the 20th century from Thomas Hunt Morgan’s laboratory (Bellen and Yamamoto, 2015). Four sets of tools were critical for mapping mutations from our X-screen: (1) chromosomal duplications and translocations that involve large segments of the X-chromosome, (2) molecularly defined deficiencies, (3) lethal transposon insertions and previously identified mutations in essential genes, and (4) a collection of transgenic fly strains carrying ∼80kb BAC (Bacterial Artificial Chromosomes) spanning the entire X-chromosome.

The general strategy of our mapping process is illustrated in Figure 1C (See Haelterman et al., 2014a for details). The first step is to identify a chromosomal duplication that rescues the lethality of the mutant of interest. Thousands of stocks are available from stock centers such as the Bloomington *Drosophila* Stock Center (BDSC) at Indiana University^3^ and *Drosophila* Genomics and Genetic Resources (DGGR) at Kyoto Institute of Technology^4^ for this purpose. In addition to classic cytologically mapped duplications, Kevin Cook and his colleagues have generated a large collection of molecularly defined X-chromosome duplications translocated to the Y chromosome [*Dp(1;Y)*] (Cook et al., 2010). By crossing individual lines to 21 duplications that cover > 90% of the fly X-chromosome, we rescued and mapped 1,305 mutants to ∼1Mb regions. These mutant lines are publicly available from the BDSC^5^ or DGGR^6^. We assembled mutants that were rescued by the same duplication into complementation groups by crossing the rescued males to mutant females. The rescued males were also used to fine map the mutations to smaller chromosomal regions by crossing them to flies carrying molecularly defined deficiencies. A tiling kit of deficiencies that cover the entire genome allows one to map lethal mutations to small regions that, on average, contain about nine genes (Parks et al., 2004; Ryder et al., 2004; Cook et al., 2012). X-screen mutant males that are rescued by a specific duplication were also crossed to flies carrying mutations in known essential genes to determine whether they were allelic or not.

If the mutation of interest complemented all of the known lethal mutations in the region of interest, we sequenced the genes within the mapped interval. During the early phase of the project, we prioritized sequencing of coding regions via Sanger sequencing. Eventually, we shifted to utilizing whole-genome sequencing (WGS) when this technology became affordable. Although several studies had utilized WGS for mapping a few chemically induced mutations in *Drosophila* prior to this work (Blumenstiel et al., 2009; Gonzalez et al., 2012), our study was the first to apply WGS to map hundreds of mutants derived from a single screen (Haelterman et al., 2014a). Once a candidate gene was identified, the final step was to show that the variant of interest was the cause of lethality as well as phenotypes observed in mutant clones. This was extremely important since chromosomes that are treated with EMS carry hundreds of potential functional variants. By crossing a transgenic fly with a genomic fragment that contains a wild-type copy of the gene of interest to the X-screen mutant fly, we tested whether the lethality and other phenotypes (e.g., ERG defects) could be rescued by this manipulation. To facilitate this process, Hugo Bellen, Koen Venken and their colleagues established ∼400 BAC transgenic lines (Venken et al., 2009, 2010) that tile the entire X-chromosome using the P[acman] technology (Venken et al., 2006). Each line contains a ∼80kb BAC inserted into a specific location of the genome using the ϕC31 integrase. If a specific BAC that covers the variant of interest rescues the lethality and phenotypes of the mutant, and there are no other functional changes identified within the ∼80kb interval, we attributed the phenotype of interest to the gene of interest. Using these methodologies, we mapped 614 mutations onto 165 genes, about half of which exhibited ERG defects. Comparison of the list of genes isolated from this screen and similar RNAi screens for developmental phenotypes revealed that there were limited overlaps (Mummery-Widmer et al., 2009; Saj et al., 2010; Oortveld et al., 2013; Yamamoto et al., 2014), suggesting that EMS screens and RNAi screens reveal complementary sets of genes.

It is important to note that although the X-screen was based on random mutagenesis, we were able to identify human homologs for 93% of the genes from the screen, a surprising enrichment compared to the rest of the genome in which ∼60% of fly protein coding genes are conserved in humans (Wangler et al., 2015). This enrichment may be due to the fact that the screen was designed to capture essential genes that regulate neuronal function and maintenance or development, which are conserved biological processes. Moreover, at the time of analysis (2014), 31% of the X-screen fly genes had at least one human homolog that was linked to a Mendelian disorder in OMIM (Online Mendelian Inheritance in Man)^7^, most (∼75%) of which exhibited neurological symptoms. The fraction of X-screen genes linked to Mendelian disease has increased to ∼50% in the past 4 years, primarily due to the rapid pace of disease gene discovery taking place in the human genomics field (Zhang, 2014; Alkuraya, 2016). In some instances, the fly study served as a catalyst to identify patients who carry deleterious variants in the human homolog of the *Drosophila* gene (Yamamoto et al., 2014; Yoon et al., 2017; Tan et al., 2018). Considering the importance of these genes to survival and nervous system function in *Drosophila*, we predict that most, if not all, conserved X-screen genes will eventually be linked to human diseases.

## Ultrastructural and Functional Characterization of Neurodegenerative X-Screen Mutants Allowed Classification of Genes That Are Critical for Neuroprotection

**FIGURE 2:**
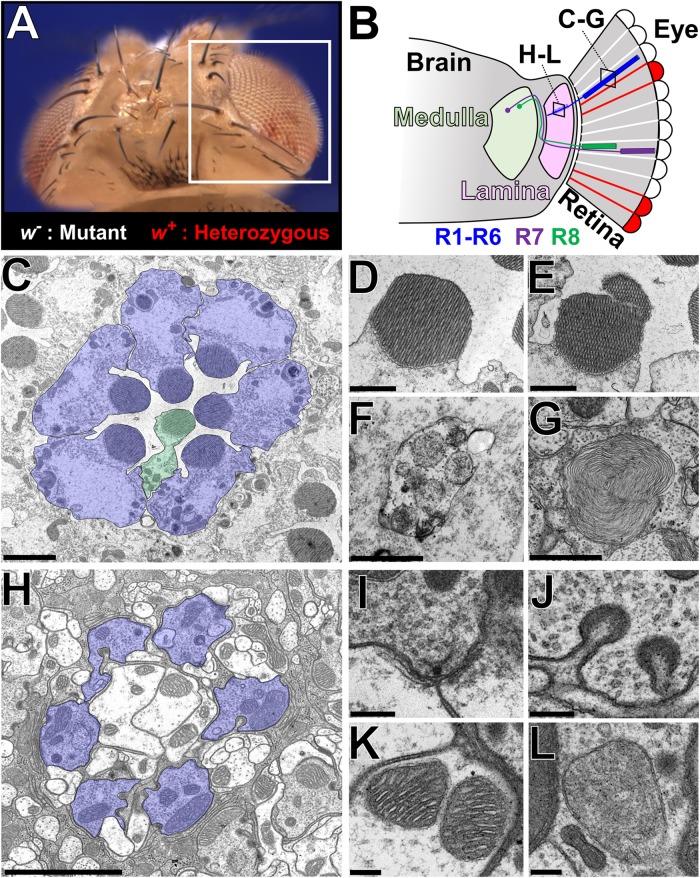
Ultrastructural analysis of the *Drosophila* visual system to assess neurodegenerative phenotypes in X-screen mutant clones. **(A)** Image of an adult *Drosophila* head that is mosaic for a mutation of interest (*y w mut^∗^ FRT19A/cl(1) FRT19A; ey-FLP/+*). The white box indicates the region depicted in **B**. **(B)** Simplified diagram of the *Drosophila* visual system. The retina is composed of ∼700 ommatidia, each containing eight photoreceptors that are surrounded by a layer of pigment cells that function as glia. Photoreceptors R1–R6 target their axons to the lamina, whereas R7 and R8 connects to the medulla. The two boxes indicate the location of TEM sections shown in **C–G** and **H–L**, respectively. **(C)** TEM image of a cross section through a single ommatidia. R1–R6 is highlighted in blue, and a R7 or R8 cell (stacked on top of one another as in **B**) is highlighted in green. **(D–G)** Higher magnification images of some subcellular structures observed in the retina; **(D)** Healthy rhabdomere, **(E)** rhabdomere undergoing fragmentation, **(F)** autophagosome, **(G)** multilamellar body. **(H)** TEM image of a cross section through a single lamina cartridge. Axon terminals of R1–R6 cells (highlighted in blue) synapse onto dendrites of large monopolar cells neurons and amacrine cells in the lamina. Each cartridge is separated by three epithelial glial cells. **(I–L)** Higher magnification images of some subcellular structures observed in the lamina; **(I)** synaptic active zone marked by a T-bar, **(J)** capitate projections, **(K)** mitochondria with normal cristae structure, and **(L)** abnormal mitochondria that lack fine cristae structure. Scale bars = 2 mm **(C,H)**, 1 mm **(D–G)**, and 200 nm **(I–L)**. TEM images were kindly provided by Lita Duraine (Howard Hughes Medical Institute, Baylor College of Medicine).

To further characterize mutants in which multiple alleles of a single gene exhibited ERG defects that worsen with age, we performed transmission electron microscopy (TEM) on eye and brain tissue from young and aged animals (Figure 2). By cross-sectioning the retina, we can visualize the cell bodies of seven out of eight photoreceptors (R1-R6 and R7 or R8) that comprise each ommatidium together with the supporting glia cells (pigment cells) that surround this structure (Figure 2C; Melnattur and Lee, 2011). Each photoreceptor possesses a large rhabdomere or a membrane rich apical structure containing the photosensitive G-protein coupled receptor Rhodopsins (Rh) (Xiong et al., 2012). Integrity of the rhabdomere and appearance of organelles such as autophagosomes and multilamellar bodies (Phillips et al., 2008) can serve as indicators of cellular health (Figures 2D–G). In this system, photoreceptor loss can be spotted by counting the number of rhabdomeres per ommatidium or by identifying degenerative structures such as vacuoles that arise from loss of an entire ommatidium. In addition, by sectioning through the lamina of the brain, one can examine lamina cartridges comprised of presynaptic axonal terminals of six photoreceptors (R1-R6) making contacts with three post-synaptic lamina large monopolar neurons and an amacrine cell (Figure 2H; Melnattur and Lee, 2011; Rivera-Alba et al., 2011). Each cartridge is surrounded by three epithelial glial cells (Edwards et al., 2012). TEM allows visualization of fine structures such as synaptic vesicles, active zones [site of synaptic vesicle release (Fouquet et al., 2009)], synaptic mitochondria, and capitate projections [a glial structure that participates in the recycling of neurotransmitters (Rahman et al., 2012)] (Figures 2I–J). The overall integrity of the cartridge architecture and morphology of subcellular organelles such as the mitochondria can serve as indicators of neurodegeneration (Figures 2K,L). Using these landmarks, we assessed whether age-dependent deterioration of ERG signals in mutant clones correlated with morphological changes in photoreceptor cell bodies or in synapses. We also determined if there are structural deficits in newly eclosed flies as a means to detect any pre-symptomatic signs of neurodegeneration.

**FIGURE 3:**
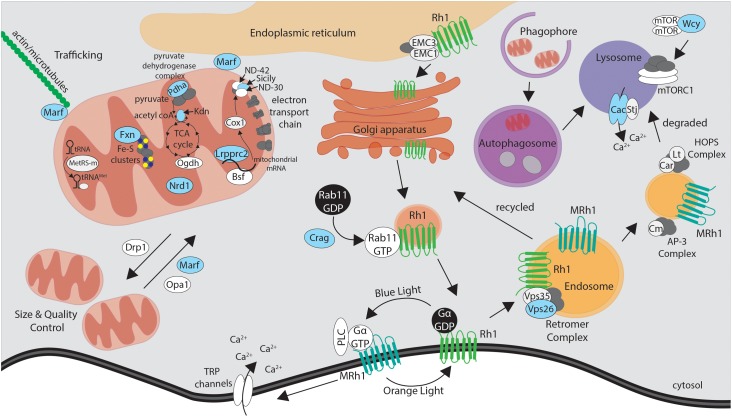
Simplified schematic of the proteins encoded by essential genes required for neuronal maintenance reviewed in this article. Mutations that affect proteins highlighted in blue were identified through the X-screen. Proteins shown in white were studied in the context of X-screen projects or by other groups in neurodegeneration in *Drosophila*. See text and Table 1 for more detail on the functions and human disease associations of individual proteins and genes.

In addition to electrophysiological, histological and ultrastructural studies of mutant tissue, we performed functional assays specific to the gene of interest to understand the molecular mechanisms underlying neurodegenerative phenotypes. Interestingly, many of the mutants were in genes functionally related to the mitochondria (Figure 3 and Table 1). Mitochondrial function can be assessed by multiple parameters such as electron transport chain (ETC) activity, reactive oxygen species (ROS), and ATP levels (Brand and Nicholls, 2011). Features such as size and number of mitochondria and morphology of the crista based on TEM can also provide indication about the health of the cell. Copy number of mitochondrial DNA and levels of mitochondrial transcripts also provides useful molecular information. Metabolites generated by the mitochondria, such as products of the Krebs cycle, can be measured through mass spectrometry-based metabolomics profiling, providing additional clues to the origins of neurodegenerative phenotypes. By combining a number of genetic, cellular, molecular, and biochemical tools, we attempted to assess what features of mitochondrial function, if any, are defective in specific mutants. In addition to genes associated with mitochondria, several genes that exhibited neurodegenerative phenotypes were linked to vesicular trafficking and autophagy (Figure 3 and Table 1). In order to understand the molecular defects related to this group of genes, we utilized immunofluorescence staining as well as biochemical assays such as western blotting, protein trafficking and turnover assays in our studies.

**Table 1:** Neuroprotective *Drosophila* genes discussed in this article and their links to human neurological diseases.

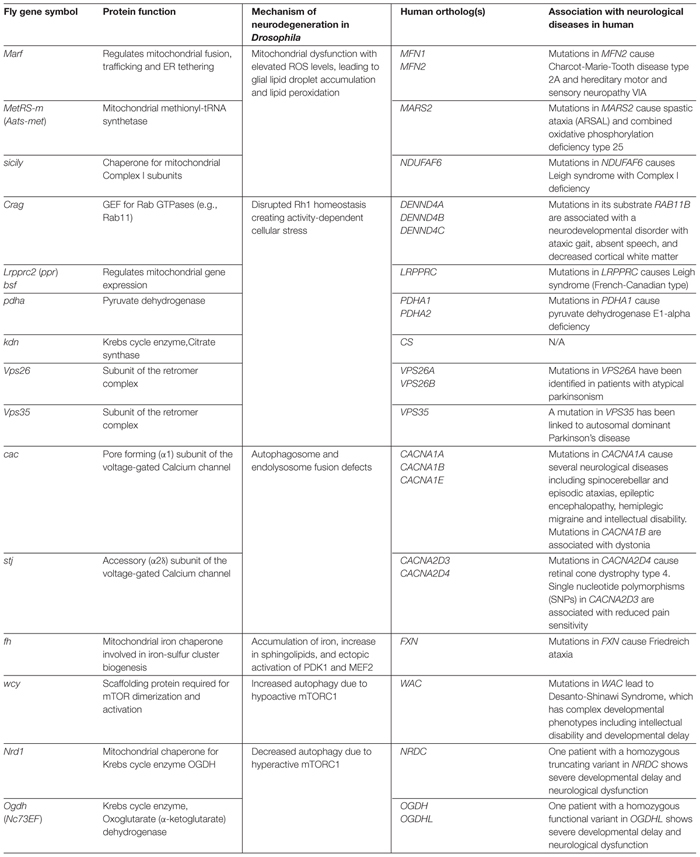

Functional studies of mutants identified from the X-screen revealed novel insights into the *in vivo* function of many genes, most of which had been linked to human diseases (Yamamoto et al., 2014). As summarized in Table 1, X-screen neuroprotective genes (genes that cause neurodegeneration upon LOF) can be classified based on the molecular mechanism that underlie their neurodegenerative phenotypes. Although vertebrate homologs of many of these genes had been studied prior to our work, systematic phenotyping and detailed *in vivo* functional studies led to discovery of novel mechanisms of neurodegeneration that are evolutionarily conserved. In the following sections, we discuss the molecular functions of neuroprotective X-screen genes and how dysfunction of these genes causes neurodegeneration, particularly in the fly visual system. We will provide links to human neurodegenerative disorders whenever applicable.

## Mitochondrial Dysfunction, Oxidative Stress and Lipid Droplets in Neurodegeneration

Mitochondria are essential to many cellular processes including metabolism, cell proliferation and cell death. Due to their high-energy demands, dynamic signal regulation and low regenerative capacity, neurons are particularly susceptible to mitochondrial dysfunction. Indeed, many mitochondrial diseases exhibit neurodegenerative phenotypes, and mitochondrial dysfunction is one of the key hallmarks associated with more common neurological disorders such as AD, PD and ALS (Lin and Beal, 2006). Animal models have made a number of links between mitochondrial pathways and neurodegenerative disease pathology (Lin and Beal, 2006; Haelterman et al., 2014b; Kawamata and Manfredi, 2017). Most genes that function in mitochondria are conserved between humans and *Drosophila*, allowing exploration of their *in vivo* functions in flies (Debattisti and Scorrano, 2013). By studying multiple genes from the X-screen and comparing their phenotypes in parallel, we identified a novel evolutionarily conserved metabolic pathway that contributes to the neurodegenerative phenotypes in mutants with high ROS. Here, we first introduce the molecular functions of the genes of interest, and discuss how comparing ultrastructural phenotypes of mutants in a systematic function led to this novel finding.

### Marf

Mitochondrial dynamics, such as fission and fusion, are required to maintain the organelle’s integrity, localization, and trafficking (Westermann, 2010; Chan, 2012). Mutations in genes that regulate mitochondrial dynamics are linked to several neurological disorders in humans. These include the mitochondrial fusion GTPases *MFN2* and *OPA1* that are linked to Charcot-Marie-Tooth Disease type 2A (OMIM: #609260, Züchner et al., 2004; Chung et al., 2006) and optic atrophy type 1 (OMIM: #165500, Delettre et al., 2000), respectively, and the mitochondrial fission GTPase *DNM1L* that causes fatal infantile encephalopathy (OMIM: #614388, Waterham et al., 2007) and optic atrophy type 5 (OMIM: #610708, Gerber et al., 2017) when mutated.

The first LOF mutations in *Drosophila Marf* (*Mitochondrial assembly regulatory factor*, ortholog of *MFN1* and *MFN2*) were isolated from the X-screen, while mutant alleles of *Drp1* (*Dynamin related protein 1*, ortholog of *DNM1L*) were established through a similar forward genetic screen on the *Drosophila* second chromosome (Verstreken et al., 2005). Mutations in *Opa1* (*Optic atrophy 1*) were generated using a transposable element-based gene deletion approach and were characterized to exhibit developmental phenotypes in the eye and male gonad (McQuibban et al., 2006; Yarosh et al., 2008). *Marf*, *Opa1* and *Drp1* are all essential genes, and LOF alleles exhibit larval lethality. Disruptions in any of these three GTPases reduces energy production and blocks mitochondrial trafficking down the motor neuron axons to the neuromuscular junction, indicating a role for mitochondria dynamics in both ATP synthesis and organelle trafficking (Verstreken et al., 2005; Sandoval et al., 2014). Interestingly, *Marf* and *Opa1* mutants exhibited defects in synaptic morphology at the neuromuscular junction, a phenotype that was not seen in the *Drp1* mutant animals. Surprisingly, this phenotype was not rescuable by re-introduction of wild-type Marf protein in neurons, muscle or both of these tissues, suggesting cell non-autonomy. Through additional tissue-specific rescue experiments, Sandoval et al. (2014) identified that the synaptic phenotype was due to loss of *Marf* function in the ring gland, the key endocrine organ responsible for controlling development, metabolism, growth, reproduction and specific behaviors. In this tissue, *Marf* is required for the synthesis/accumulation of lipid droplets and mitochondrial-ER tethering, both of which are critical for timely synthesis of a key steroid hormone, ecdysone. Since *Marf* is orthologous to both *MFN1* and *MFN2*, we assessed whether these human genes can rescue the loss of *Marf* in *Drosophila*. Although MFN2 was sufficient to rescue the ER tethering and lipid droplet synthesis defects in the ring gland, both MFN1 and MFN2 were required to fully rescue the synaptic morphology, ecdysone synthesis, and mitochondrial trafficking defects seen in *Marf* knockdown animals (Sandoval et al., 2014). This suggests that human MFN1 and MFN2 have distinct molecular functions, both of which being present in the single fly Marf protein. Indeed, studies using single and double knockout mice for *Mfn1* and *Mfn2* revealed that the two genes have overlapping as well as distinct molecular functions (Cipolat et al., 2004; de Brito and Scorrano, 2008; Naon et al., 2016). While it is difficult to pinpoint the organ that is truly homologous to the ring gland in vertebrates, it is interesting to note that *OPA1* and *MFN2* seems to have distinct roles in vertebrate steroidogenesis (Issop et al., 2013).

### MetRS-m

With only 13 protein-coding genes remaining in the mitochondrial genome in *Drosophila* and humans, most of the genes encoding proteins that function in mitochondria were translocated from the mitochondrial genome to the nuclear genome after establishment of the endosymbiotic relationship between the ancestral eukaryotic cell and the symbiotic bacteria (Leister, 2005). These include several mitochondrial tRNA synthetases that conjugate specific amino acids to their corresponding tRNA. Mutations in many of these genes lead to human diseases with neurological presentation (Konovalova and Tyynismaa, 2013). Bayat et al. (2012) identified that *MetRS-m* [*mitochondrial Methionyl-tRNA synthetase*, also referred to as *Aats-met*, human ortholog: *MARS2*] is required for neuronal development and maintenance in *Drosophila* by characterizing two missense alleles isolated from a third chromosome forward genetic screen. *MetRS-m* mutant clones show progressive photoreceptor degeneration accompanied by abnormal mitochondrial morphology. Upon closer examination, they found reduced cell proliferation in developing tissues, disrupted activity of Complex I of the ETC, and increased ROS. Previously, a human neurological disease found in the French-Canadian population named ARSAL (Autosomal Recessive Spastic Ataxia with Leukoencephalopathy, also known as autosomal recessive spastic ataxia type 3, OMIM: #611390) was mapped to a region containing *MARS2* (Thiffault et al., 2006). However, sequencing of the locus did not reveal obvious functional variants in *MARS2*. The *Drosophila* data was communicated to the team of human geneticists that had been trying to map the ARSAL gene, which prompted them to perform additional molecular analysis of the locus. This effort revealed that ARSAL patients carried complex genetic rearrangements involving partial duplications, deletions and inversions of *MARS2*, leading to an overall reduction in MARS2 levels. Furthermore, fibroblasts from ARSAL patients exhibited a number of defects observed in *MetRS-m* mutant flies, including reduced cell proliferation, reduced Complex I activity, and increased ROS (Bayat et al., 2012). This study is an early example in which dialogs between *Drosophila* biologists and human geneticists were influential in identifying the cause of a rare human disease. Mutations in other mitochondrial tRNA synthetases and related genes have also been identified in *Drosophila* (*ArgRS-m*, *GatA*) (Liao et al., 2006), providing tools for the *in vivo* study of this gene family.

### Sicily

The mitochondrial ETC, composed of Complexes I-IV, is critical for ATP synthesis. As a byproduct, the ETC generates ROS. Disruption of the ETC, particularly the dysfunction of Complex I, not only reduces the amount of energy (ATP) produced in the cell, but often leads to a significant increase in ROS production. This ROS consists of highly reactive compounds such as H_2_O_2_ and OH^-^ that, when uncontrolled, can lead to DNA and protein damage as well as lipid peroxidation (Fritz and Petersen, 2013; Niedzielska et al., 2016). Moreover, alterations in Complex I activity and high ROS production are hallmarks of mitochondrial dysfunctions linked to a number of neurodegenerative disorders in humans (Morris et al., 1996; Manczak et al., 2004). Proper ETC complex activity requires all nuclear-encoded protein subunits to be made properly, transported into the mitochondria, and assembled with the mitochondria-encoded subunits into large complexes. Although the molecular structure of the ETC complexes have been elucidated in atomic resolution (Letts et al., 2016; Wu et al., 2016), identification of factors required for the assembly of these large protein complexes is still in progress. From the X-screen, we identified alleles of a previously uncharacterized gene that is orthologous to *NDUFAF6* (*NADH dehydrogenase Complex I, assembly factor 6*) and named it *sicily* (*severe impairment of Complex I with lengthened youth*) based on mutant phenotypes (Zhang et al., 2013). Mutations in human *NDUFAF6* causes Leigh syndrome with mitochondrial Complex I deficiency (OMIM: #256000, Pagliarini et al., 2008; Kohda et al., 2016). Interestingly, loss of *sicily* in flies show a number of features that are observed in patients with Leigh syndrome, such as ETC deficiency, an increase in ROS and progressive loss of neurons. Prior to this work, a study using cultured cells and patient tissues reported that NDUFAF6 is required for the assembly of Complex I (Pagliarini et al., 2008), but its precise molecular role was obscure. Using genetically tagged constructs, Zhang et al. (2013) found that Sicily localizes to both the cytoplasm and the mitochondria, and interacts with key Complex I subunits ND-42 (human ortholog: *NDUFA10*) and ND-30 (human ortholog: *NDUFS3*) specifically in the cytoplasm. Based on additional genetic and biochemical data, Zhang et al., concluded that Sicily acts as a co-chaperone for Hsp90 to stabilize Complex I proteins prior to their entry into the mitochondria. Together with insights obtained from other fly genes whose human orthologs are also linked to Leigh syndrome such as *ND-42* (Burman et al., 2014) and *Surf1* (human ortholog: SURF1, Da-Rè et al., 2014), we now have a better understanding of this disorder at the molecular, cellular and organismal level.

### ROS and Lipid Droplets

Mutations in mitochondria-related genes discussed thus far have provided insights into the function of individual genes and proteins. By comparing the phenotype across multiple mitochondrial mutants from the X-screen, we were able to identify an unrecognized role of lipid droplets in neurodegeneration (Liu et al., 2015). During the study of *Marf*, *MetRS-m* and *sicily* mutant clones in the retina using TEM, Sandoval, Bayat, and Zhang noticed that there was a peculiar phenotype that had not been reported in the literature. In 3-day post-eclosion animals, which is still a pre-symptomatic stage in these mutants, we observed an enlargement of pigment cells, which function as glia for photoreceptors. Pigment cell expansion was caused by accumulation of lipid droplets, intracellular organelles that store neutral lipids and cholesterol (Welte, 2015). Interestingly, in aged animals when photoreceptors were undergoing structural deterioration, lipid droplets were absent. This suggested that lipid droplets accumulate transiently prior to the onset of neurodegeneration. Liu et al. (2015) assessed if this was a common phenomenon in mutants affecting the mitochondria. Interestingly, while knockdown of *ND-42* and *Parkin* (E3 ligase involved in mitochondrial quality control) showed similar lipid droplet accumulation, mutant clones for *Pink1* (kinase involved in mitochondrial quality control) and *Drp1* did not exhibit this defect, suggesting that glial lipid droplet accumulation was not a general phenomenon. The number of lipid droplets correlated with the amount of ROS that was observed in the affected tissues, suggesting a link between ROS and lipid droplet accumulation. We further found that ROS generated in the photoreceptors triggers activation of JNK (c-Jun N-terminal Kinase) signaling as well as SREBP (Sterol Regulatory Element-Binding Protein), a transcription factor that functions as the master regulator of lipogenesis (Dobrosotskaya et al., 2002; Kunte et al., 2006). The lipids generated by downstream target genes of SREBP are transferred from photoreceptors to pigment cells, leading to the formation of lipid droplets in a cell non-autonomous manner. These lipid droplets by themselves are not toxic and most likely play neuroprotective roles (Bailey et al., 2015; Van Den Brink et al., 2018). However, when these lipids are peroxidated by ROS, this stresses the cells and eventually leads to degeneration of both cell types. Introducing antioxidants, inhibiting JNK or SREBP, or knocking down lipogenesis enzymes in the photoreceptor cells reduces glial lipid droplet accumulation and delays the onset of neurodegeneration in these mutants (Liu et al., 2015).

Although neural lipid accumulations had been reported in several neurodegenerative mouse models (Mato et al., 1999; Wang et al., 2002), there was little evidence for these molecules having a direct role in neurodegeneration. This is likely because many studies on mice and human autopsy brains have studied neurons that have undergone or are actively undergoing neurodegeneration, whereas lipid droplet accumulation is only observed in pre-symptomatic stages in both flies and mice. Liu et al. (2015, 2017) found that the mice with defects in the ETC Complex I (Ndufs4-/-, Kruse et al., 2008; Quintana et al., 2012) as well as in a mouse neuron-astrocyte cell co-culture system with ROS induction via rotenone exhibit glial (astrocyte, microglia) lipid droplet accumulation. Using this co-culture model and *Drosophila*, Liu et al. (2017) determined that lactate plays a critical role in glial lipid droplet formation. The shuttling of lactate from glia to neurons via monocarboxylate transporters provides a substrate for lipid synthesis, which is then shuttled back from neurons to glia for lipid droplet formation via fatty acid transporters and apolipoproteins (Liu et al., 2017). By focusing on Apolipoprotein E (APOE), they found that this protein mediates the transport of lipids from glia to neurons in culture. Although the direct ortholog of *APOE* is absent from the fly genome, human APOE was able to substitute for the loss of *GLaz* (*Glial Lazarillo*), a *Drosophila* apolipoprotein orthologous to human *APOD*. Common variants in *APOE* are known to be associated with the risk of late-onset AD (Kim et al., 2009; Yu et al., 2014). One copy of *APOE*^*ε*4^ variant increases the chance of developing AD (odds ratio: 4.2) compared to the most common *APOE*^*ε*3^ allele, whereas *APOE*^*ε*2^ variant is considered to be neuroprotective (odds ratio: 0.7) (Sando et al., 2008). Interestingly, while human APOE^*ε*2^ and APOE^*ε*3^, were capable of functionally replacing the *Drosophila GLaz* in lipid droplet transport, APOE^*ε*4^ was not able to perform this task. Furthermore, the *GLaz* mutant files expressing human APOE^*ε*4^ showed signs of neurodegeneration that were not seen in animals expressing APOE^*ε*2^ and APOE^*ε*3^ (Liu et al., 2017). Overall, this suggests that *APOE*^*e*4^ has reduced capacity to transport lipids between glia and neurons, thus making those flies susceptible to neurodegeneration in the presence of elevated ROS. Whether this function of APOE contributes to pathogenesis of AD and other neurodegenerative diseases in humans awaits further exploration.

## An Unanticipated Link Between Rhodopsin Trafficking, Mitochondria and Endolysosomal Pathway

Rh are evolutionarily conserved light-sensing G protein-coupled receptors expressed in photoreceptors and a few additional cell types (Alvarez, 2008). Some Rh are used for color vision (e.g., fine-tuned to certain wavelengths), whereas others are primarily responsible for movement detection and night vision (e.g., highly sensitive to photons). Mutations in genes that encode Rh or factors that regulate Rh synthesis, maturation, degradation or activity often lead to improper development or maintenance of photoreceptors and to retinal degeneration. In humans, there are more than 100 different mutations in Rh (human gene symbol: *RHO*) that cause progressive eye diseases known as retinitis pigmentosa (OMIM #268000). Due to evolutionary conservation and powerful genetic methodologies, *Drosophila* has been used extensively to understand Rh biology and to identify mutations that cause retinal degeneration.

Rh1, the primary Rh in *Drosophila* photoreceptors encoded by the *ninaE* (*neither inactivation nor afterpotential E*) gene, is synthesized and folded in the ER (Baker et al., 1994; Xiong and Bellen, 2013). Mature Rh1 consists of a 7-transmembrane protein (Opsin) which is covalently bound to its cofactor retinal, a vitamin A-based retinaldehyde chromophore. As Rh1 moves through the secretory pathway through the Golgi apparatus, it is post-translationally modified prior to reaching the plasma membrane. Upon absorbing a photon of light, Rh1 changes its conformation to become active. Activated Rh1, referred to as Metarhodopsin 1 (MRh1), triggers a phototransduction cascade through heterotrimeric G proteins, opening up TRP (Transient Receptor Potential) channels allowing Ca^2+^ entry and photoreceptor depolarization. Although the downstream components of the *Drosophila* phototransduction cascade are different from mammalian cone and rod photoreceptor cells, a homologous pathway operates in a subset of retinal ganglion cells that are intrinsically photosensitive and express Melanopsin, a mammalian Rh that shows the highest homology to *Drosophila* Rh1 (Pickard and Sollars, 2011).

Since Rh1 is highly abundant within R1-R6 photoreceptors cells, altered trafficking or accumulation of Rh1 due to improper synthesis, maturation or degradation can create cellular stress and disrupt the function and overall structure of photoreceptors. Aberrant accumulation of Rh causing a ‘traffic jam’ and cytotoxicity due to altered Rh activity are the two primary causes of retinal degeneration in mammalian species. For example, the most frequent mutation found in human *RHO* that causes retinitis pigmentosa is a missense variant (p.P23H) that is inherited in an autosomal dominant fashion (Dryja et al., 1990). When the analogous mutation was introduced into *Drosophila Rh1* (p.P37H), Rh1 accumulated in the ER and caused an age-dependent photoreceptor degeneration phenotype (Galy et al., 2005). Pathogenic mutations in genes that regulate RHO trafficking such as *CRB1* (*Crumbs* in fly) also cause retinitis pigmentosa in humans and have also been shown to mediate retinal degeneration in *Drosophila* (O’Tousa, 1992; Li et al., 1994; den Hollander et al., 1999; Pellikka and Tepass, 2017). Historically, most of the studies on Rh1 homeostasis in flies have focused on non-essential genes that have specialized functions in the eye (Wang and Montell, 2007; Xiong and Bellen, 2013). In this section, we highlight several essential genes identified from the X-screen that play critical roles in Rh1 homeostasis and discuss how this relates to our understanding of human neurological diseases beyond retinal degeneration.

### Crag

*Drosophila* Rh1 is converted into MRh1 upon absorbing a photon that is in the blue wavelength (Kiselev and Subramaniam, 1994). While MRh1 can be converted back to Rh1 through the absorption of a second photon with a different wavelength (orange light) (Minke, 2012), a certain fraction of MRh1 along with some non-activated Rh1 becomes internalized and degraded through the endolysosomal pathway upon light stimulation (Satoh and Ready, 2005; Orem et al., 2006). This means that continuous supply of Rh1 is required even after photoreceptor development has completed. The transport of Rh1 from the *trans*-Golgi network to the rhabdomeric membrane requires the small GTPase Rab11 (Satoh et al., 2005). Rab GTPases require guanine nucleotide exchange factors (GEF) for activation, and the gene product of *Crag* (*Calmodulin binding protein related to Rab3 GDP/GTP exchange protein*) was found to play this role in photoreceptors post-developmentally (Xiong et al., 2012). Xiong et al. (2012) found that in the absence of *Crag*, there is an activity- and age-dependent decline in photoreceptor function and structural integrity, shown via ERG and TEM analyses, respectively. This coincided with accumulation of Rh1 in intracellular vesicles, similar to what was observed upon knockdown of *Rab11*. Crag is capable of acting as a GEF for Rab11 *in vitro*, and expression of a constitutively active Rab11 that does not depend on its endogenous GEF can suppress the loss of *Crag* to some extent. Similar to fly Crag, expression of a human DENND4A, one of three human Crag orthologs, can rescue the defects in the mutant fly eye, demonstrating that the function of this protein is evolutionarily conserved. So far, no Mendelian disease has been associated with *DENND4A* or other paralogs (*DENND4B, DENND4C*); however, *de novo* missense mutations in *RAB11B*, one of two *Rab11* human orthologs, cause a neurodevelopmental disorder with ataxic gait, absent speech, and decreased cortical white matter (OMIM #617807). These patients also exhibit intellectual disability and visual impairments (Lamers et al., 2017). Considering that Rab11 functions as a key trafficking regulator for endocytic recycling, neurological phenotypes seen in the patients are likely caused by defective trafficking of transmembrane proteins critical for neurodevelopment.

Another example of essential genes required for Rh1 trafficking in flies that have been linked to a neurodegenerative disease in humans are genes that encode subunits of the ER membrane protein complex (EMC). EMC1 and EMC3 are required for stable expression of Rh in addition to other multi-pass transmembrane proteins and cause photoreceptor degeneration when lost in flies (Satoh et al., 2015). In humans, *EMC1* mutations cause an autosomal recessive disease characterized by cerebellar atrophy, visual impairment and psychomotor delay (OMIM #616875, Harel et al., 2016a). Although the time-course of visual impairments has not been documented, patients exhibited a clear progressive cerebral and cerebellar atrophy phenotype, indicating that this is a degenerative disorder affecting multiple brain regions. The discovery of Crag as a GEF for Rab11, and EMC proteins as stabilization factors of transmembrane proteins including Rh1 provides a starting place to understand human neurological diseases that may be caused by mistrafficking of transmembrane proteins expressed in the nervous system.

### *Lrpprc2, kdn, pdha*, and *bsf*

The study of Rh in *Drosophila* can also be used to dissect unknown mechanisms of neurodegenerative diseases that affect tissues other than the eye. Many neurodegenerative disorders are complex and likely have multiple molecular mechanisms contributing to the disease phenotype. For example, Leigh syndrome (OMIM #256000) is an early-onset progressive neurodegenerative disorder that affects a number of different areas of the central nervous system (CNS) as well as a many other organ systems, leading to death in early infancy in many cases (Leigh, 1951; Dahl, 1998). Mutations in more than 70 different genes cause Leigh syndrome, and most are linked to disruption of mitochondrial functions, typically altering the ETC (Lake et al., 2016). Most of these disease-causing mutations are thought to cause an increase in ROS, which in turn leads to neurodegeneration (Koopman et al., 2008; Quintana et al., 2010; Hayashi and Cortopassi, 2015). As mentioned previously, we identified a number of genes from the X-screen that are linked to mitochondrial function, some of which are linked to Leigh syndrome (Table 1). While defects in some Leigh syndrome-associated genes do indeed show increased ROS, including *sicily*, disruption of the fly ortholog of *LRPPRC* [*Leucine-rich PPR motif-containing protein, mitochondrial*, *Lrpprc2* (also referred to as *ppr*)] did not result in a significant increase in ROS levels despite mutant photoreceptors undergoing neurodegeneration (Jaiswal et al., 2015).

Mutations in human *LRPPRC* cause an autosomal recessive French Canadian-type Leigh syndrome (OMIM#220111, Morin et al., 1993). By studying the cause of the degeneration seen in *Lrpprc2* mutant photoreceptors, Jaiswal et al. (2015) unexpectedly found that the neurodegeneration phenotype is caused by a disruption in Rh1 homeostasis. In the absence of *Lrpprc2*, there is a significant increase in Rh1 found in intracellular vesicles that is unable to cycle to the plasma membrane or undergo degradation. Interestingly, the degenerative phenotype can be suppressed by raising the flies in the dark (suppresses photoreceptor activation by light) or by raising the flies with low vitamin A food (reduces Rh1 synthesis), suggesting that the neurodegeneration is dependent on Rh1 activity. Loss of *Lrpprc2* caused defects in efficient processing or stabilization of most mitochondrial RNAs (mtRNAs), leading to reduced ATP synthesis without obvious increases in ROS. Furthermore, other mutants that exhibited reduced levels of ATP without an increase in ROS levels, such as *knockdown* (*kdn*, encodes Citrate Synthase) and *pyruvate dehydrogenase* (*pdha*), showed similar phenotypes (Jaiswal et al., 2015). Interestingly the human ortholog of *pdha*, *PDHA1*, is linked to pyruvate dehydrogenase E1-alpha deficiency (OMIM #312170, Brown et al., 1994), an X-linked disease that manifests with severe metabolic and neurologic symptoms. Since PDHA1 generates acetyl-CoA that is critical for the Krebs cycle, aerobically active cells including neurons may be more sensitive to its loss. Exploration of the cellular impact upon loss of *PDHA1* in neurons, particularly on trafficking of transmembrane proteins, may help understand some of the nervous system abnormalities in this complex disorder.

It is interesting to note that *Lrpprc2* has a paralog in the fly genome named *bsf* (*bicoid stability factor*) that is also orthologous to human *LRPPRC* (Sterky et al., 2010). This gene has been studied in the context of mRNA stability during early development (Mancebo et al., 2001) and was also shown to be necessary for stabilization of a subset of mtRNAs through gene knockdown studies (Bratic et al., 2011). Jaiswal et al. (2015) found that complete loss of *bsf* leads to severe mtRNA processing defects similar to *Lrpprc2* mutants, suggesting that the two genes have overlapping biological functions but are not redundant. Studies of genes that are duplicated in *Drosophila* compared to humans allow us to begin to understand how genes maintain, lose, or gain functions during evolution, and may provide insights to understanding how genes that are duplicated in humans but not in flies (e.g., *Marf* and *MFN1*/*MFN2*) have evolved over time.

### *Vps26* and *Vps35*

Studies of Rh1 trafficking have also provided insights into common neurodegenerative diseases such as PD. From the X-screen, we isolated alleles of *Vps26 (Vacuolar protein sorting 26)* that exhibited Rh1 trafficking defects and degeneration of photoreceptors (Wang et al., 2014). Vps26 forms the retromer complex with Vps35 and Vps29 to retrieve proteins from endosomes before they are degraded in lysosomes (Haft et al., 2000). Wang et al. (2014) showed that similar to *Vps26*, loss of *Vps35* in photoreceptors also causes a degenerative phenotype, indicating that the retromer complex recycles Rh1. Without proper recycling, Rh1 accumulates in late endosomes, creating stress to the endolysosomal pathway. Similar Rh1 trafficking defects that cause severe endosomal accumulation of Rh1 have been reported in *norpA* mutations [*no receptor potential A*, encoding a Phospholipase C (PLC)], in genes that regulate lysosomal biogenesis [e.g., *carmine (cm)* encoding an AP-3 (Adaptor Protein-3) complex subunit], and in genes that function in endolysosomal fusion [e.g., *light* (*lt*) and *carnation* (*car*) encoding a HOPS (HOmotypic fusion and Protein Sorting) complex subunit] (Chinchore et al., 2009). Interestingly, overexpression of retromer proteins in *norpA* or *cm* mutant cells can suppress photoreceptor degeneration, indicating that increase in retromer function can relieve the endolysosomal stress in these mutant photoreceptors. The human genome contains two genes that correspond to the single *Vps26* gene in flies, *VPS26A* and *VPS26B*. Expression of either one of these genes can effectively rescue the *Vps26* mutant phenotype (Wang et al., 2014), suggesting that both human proteins have maintained the ancestral function of Vps26.

In mice, *Vps35* gene expression is highly enriched in Melanopsin-expressing intrinsically photosensitive retinal ganglion cells that regulate pupillary light reflex (Liu et al., 2014; Wang et al., 2014). Since the phototransduction cascade of this cell type is homologous to that of *Drosophila* (Pickard and Sollars, 2011), retromer may play a role in non-visual light dependent phototransduction cascade in mammals. Moreover, mutations in *VPS26A* have been associated with atypical parkinsonism (McMillan et al., 2016) and a missense mutation (p.D620N) in *VPS35* has been linked to autosomal dominant PD (Vilariño-Güell et al., 2011; Zimprich et al., 2011). More recently through experiments in *Drosophila*, *PLA2G6*, a gene linked to PD and other neurodegenerative diseases (OMIM #256600, #610217, #612953), was shown to function as a facilitator of retromer function (Lin et al., 2018). Interestingly, variants linked to lysosomal storage disorders increase the risk of developing PD (Robak et al., 2017), indicating that defects in the endolysosome pathway may predispose individuals to PD. Together, these studies highlight the importance of vesicular trafficking and the endolysosomal pathway in neurodegeneration and shows that *Drosophila* is a useful model to understand the role of this process in PD and related disorders.

## Bridging the Link Between Frataxin, Iron Accumulation and Neurodegeneration

Iron in the form of Fe^2+^ or Fe^3+^ is a critical cofactor for many proteins involved in diverse cellular processes, including cell proliferation, DNA synthesis and, notably, mitochondrial respiration. For example, each of the ETC complexes have one or more iron-sulfur clusters that are required for their proper function (Xu et al., 2013). In the nervous system, iron deficiency has been associated with diseases including restless leg syndrome and cognitive dysfunction (Beard and Connor, 2003). In addition, excessive iron accumulation in specific regions of the brain is a hallmark of genetic neurodegenerative diseases known as NBIA (Neurodegeneration with Brain Iron Accumulation, Dusek and Schneider, 2012; Arber et al., 2016). Moreover, although not as robust as NBIA, iron accumulation in certain regions of the CNS have also been reported in patients with more common neurodegenerative disorders, including AD, PD, HD, ALS, and MS (Anderson et al., 2008; Stephenson et al., 2014). While the accumulation of iron has not been proven as the sole cause of pathogenesis in these disorders, the fact that iron rich centers in the brain such as the basal ganglia are often more susceptible in neurodegenerative diseases suggests their potential involvement (Aylward et al., 2004; Biasiotto et al., 2016; Giguère et al., 2018). The focal hypothesis for iron-induced cytotoxicity is via ROS (Batista-Nascimento et al., 2012; Núñez et al., 2012). Free iron can lead to elevated levels of ROS by Fenton chemistry, the process whereby iron cycles between two states, Fe^2+^ and Fe^3+^, producing free radicals as a side product (MacKenzie et al., 2008). While oxidative stress through ROS can certainly contribute to progression of neurodegeneration as discussed earlier, recent evidence in flies and mice suggest that iron accumulation, at least in the context of *Frataxin* (*FXN*) mutations, can cause neurodegeneration through a distinct pathway (Chen et al., 2016a,b).

### fh

Friedreich’s ataxia (FRDA, OMIM #229300, Campuzano et al., 1996) is a progressive neurodegenerative disorder caused by mutations in *FXN*. *FXN* encodes an evolutionarily conserved mitochondrial protein required for iron-sulfur cluster assembly. LOF studies in multiple species show reduced mitochondrial ETC activity, which was proposed to lead to increase levels of ROS (Al-Mahdawi et al., 2006; Anderson et al., 2008). However, Chen et al. (2016a,b) recently showed that neurodegeneration via loss of *FXN* was not mitigated by reducing ROS levels, at least in *Drosophila* and mouse models of FRDA. Instead, LOF of FXN in flies [encoded by the *frataxin homolog* (*fh*)] and mice (*Fxn*) showed massive accumulation of iron in the CNS, which led to an increase in ceramide and sphingolipid levels. Accumulation of these lipids led to ectopic activation of Pdk1, a kinase, and Mef2, a transcription factor, causing neuronal loss. Tissue from FRDA patients exhibited elevated level of sphingomyelin and activated PDK1 (Chen et al., 2016a), suggesting that this pathway is indeed ectopically activated in FRDA patients. Neurodegenerative phenotypes of *fh* mutants can be partially suppressed by inhibiting components of this pathway in *Drosophila*; this includes reducing dietary iron as well as genetic manipulations reducing sphingolipid synthesis, Pdk1 or Mef2. Ameliorating this pathway in flies, however, did not completely rescue the neurodegenerative phenotypes, indicating that other factors also contribute to the degeneration in *fh* mutant photoreceptors. Interestingly, combined treatment of iron reduction along with reduction of endolysosomal stress caused by Rh1 trafficking defects also found in *fh* mutant cells due to reduced ATP levels showed a near complete rescue (Chen et al., 2016b). These data in flies suggest that multiple cellular defects may need to be addressed together with iron-mediated toxicity in order to fully alleviate the symptoms of FRDA.

## Autophagy, an Intersection of Multiple Paths to Neurodegeneration in *Drosophila* and Humans

Autophagy typically refers to macroautophagy, a cellular process whereby double-membrane structures called autophagosomes engulf cytoplasmic proteins and organelles to promote their degradation (Ohsumi, 2014). Autophagy initiates by the fusion of vesicles, derived from multiple sources (e.g., ER, Golgi, plasma membrane), into a flattened phagophore. This autophagophore elongates and engulfs target organelles and protein complexes that are destined for degradation, forming an autophagosome. This autophagosome can fuse with late endosomes/multivesicular bodies to form amphisomes, and further fuse with lysosomes to allow breakdown and degradation of their contents. This process is critical for cells to recycle nutrients such as amino acids during periods of starvation and to clear out potentially harmful protein aggregates, damaged organelles and pathogens upon cellular stress and infections (Martini-Stoica et al., 2016). While autophagy is active in practically all cells, it has been well-studied in the context of many neurodegenerative disorders including diverse proteinopathies (e.g., AD, PD, SCA, HD prion diseases) and lysosomal storage disorders (e.g., Batten disease, Niemann-Pick disease) (Levine and Kroemer, 2008; Frake et al., 2015; Menzies et al., 2017). Understanding how autophagy participates in the maintenance of neuronal health, and how neurons degenerate when this pathway becomes misregulated will help elucidate mechanisms of diverse neurodegenerative disease pathology, potentially providing targets to develop strategies for treatment and a cure (Guo et al., 2018).

One difficulty in studying the precise role of autophagy during neural maintenance in the human brain is the fact that autophagy is used in practically all cells. Thus, many of the genes involved in autophagy likely cause a multitude of phenotypes that are difficult to segregate from cell autonomous phenotypes in neurons. Core components of autophagy (e.g., *ATG* genes) are conserved from yeast to humans, making model organisms excellent tools to study autophagy and its related genes *in vivo*. Mutations in many of the core autophagy genes lead to neurodegenerative phenotypes in flies, and mutations in orthologous genes are often associated with Mendelian neurodegenerative disorders in humans (Kim et al., 2017). Moreover, over-expression based studies that assess the effects of human proteins prone to aggregation (e.g., Tau, α-synuclein, Aβ) in the fly eye and nervous system have helped to identify functional modulators of autophagy (Ravikumar et al., 2004; Ling et al., 2009; Dinter et al., 2016). Here we discuss several X-screen related genes that exhibited defects in autophagy regulation, and emphasize how these studies have provided novel insights into human neurological disorders including discoveries of new human disease entities.

### cac

Voltage-gated calcium channels (VGCC) are pore-forming protein complexes that selectively mediate Ca^2+^ entry into cells upon membrane depolarization. In the nervous system, VGCC function has been studied primarily in synaptic transmission. VGCC localizes to the synapse and becomes activated upon transmission of action potentials. Ca^2+^ entry through the VGCC triggers the fusion of synaptic vesicles with the plasma membrane through activation of a Ca^2+^ sensor, Synaptotagmin, and SNARE (Soluble NSF attachment protein receptor) proteins. Hence, loss of synaptic transmission is the major phenotype seen in mutants that affect VGCC function in *Drosophila* (Dickman et al., 2008; Hou et al., 2008). In mice, mutations in genes that encode VGCC (Ca_v_2.1) subunits *Cacna1a* and *Cacna2d2* cause neurodegeneration, ataxia and epilepsy (Felix, 2002). Interestingly, *Cacna1a* null mice exhibit little change in excitatory synapse transmission (Jun et al., 1999), suggesting that the VGCC may play other important roles in neuronal development and maintenance.

In Tian et al. (2015), we reported an evolutionarily conserved role for VGCC in autophagy. Mutations in *cacophony* (human ortholog: *CACNA1A*) and *straightjacket* (*stj*, ortholog: *CACNA2D1-4)*, which encode the pore forming subunit and accessory subunit of the *Drosophila* VGCC, respectively, exhibited progressive degeneration of photoreceptor terminals accompanied by dramatic accumulation of autophagic vesicles. In addition to their plasma membrane localization, we determined that VGCC localizes to lysosomes and stimulates the fusion of autophagic vesicles and lysosomes. Moreover, *Cacna1a* and *Cacna2d2* mutant mice showed similar lysosomal dysfunction and neurodegeneration in the cerebellum, demonstrating a conserved role (Tian et al., 2015). In humans, mutations affecting VGCC cause a spectrum of neurological disorders. *CACNA1A* patient can present with SCA type 6 (SCA6, OMIM #183086, Zhuchenko et al., 1997), episodic ataxia type 2 (EA2, OMIM #108500, Denier et al., 2001), familial hemiplegic migraine (FHM, OMIM #141500, Ducros et al., 2001), or early infantile epileptic encephalopathy type 42 (EIEE42, OMIM #617106, EPI4K Consortium et al., 2013). Furthermore, there are associations between *CACNA1A* and congenital ataxia as well as intellectual disability (Blumkin et al., 2010). This large spectrum of disorders is thought to stem from differences in the nature of the mutations, such as polyglutamine expansions seen in SCA6 and gain-of-function mutations in FHM. However, the newly identified role of VGCC in autophagy suggests that this spectrum could also emanate from different molecular functions of VGCC (e.g., synaptic transmission versus autophagosome-lysosomal fusion defects) that are affected by different mutations. Understanding how specific mutations relate to the function of this channel *in vivo* will be critical for understanding the resulting diseases. Recent work combining human whole-exome sequencing (WES) with variant function studies using mammalian cells (Tottene et al., 2002; Wappl et al., 2002) or *Drosophila* models (Luo et al., 2017) led to understanding of the functional impact of several missense variants in *CACNA1A* found in patients with neurological diseases. Such efforts can be expanded to additional patient cohorts with potential functional variants in VGCC genes to help direct therapeutic interventions.

### wcy

Autophagy can be controlled through several different regulatory pathways, including AMP-activated protein kinase (AMPK, a sensor of energy within a cell), Eukaryotic initiation factor 2α (eIF2α, regulates protein translation), Protein kinase A (PKA, cAMP-regulated protein kinase), and stress response molecules (e.g., ER stress related proteins, JNK signaling pathway proteins, p53). One of the best-studied regulators of autophagy is mTOR (mechanistic Target of Rapamycin). mTOR functions in two distinct protein complexes, mTORC1 and mTORC2, to regulate autophagy and other aspects of cellular metabolism and protein homeostasis. Since mTOR’s activity is regulated by environmental cues such as the nutrition state of the cell or organism and growth factor signaling, it can be thought of as a molecule that balances the appropriate level of autophagy in a given cell based on intrinsic and extrinsic cues (Parzych and Klionsky, 2014; Frake et al., 2015). Interestingly, dysregulation of mTOR signaling has been implicated in neurodegenerative disorders as well as neurodevelopmental and neuropsychiatric diseases including autism spectrum disorders (Lipton and Sahin, 2014; Tang et al., 2014).

From the X-screen, we identified a novel regulator of mTOR signaling called *wacky* (*wcy*, human ortholog: *WAC*) that exhibited neurodegeneration phenotypes in the retina and the brain when mutated (David-Morrison et al., 2016). By combining *Drosophila* biology with mammalian cell-based assays, David-Morrison et al. (2016) determined that Wcy/WAC facilitates the activity of mTORC1 by assembling a protein complex that is required to dimerize mTOR. Since this dimerization is an important step for mTORC1 activation, the resulting phenotypes seen in *wcy* mutants are similar to the loss of mTOR (encoded by the *Tor* gene in flies), leading to increased autophagy. Similar to *Tor* mutants, *wcy* mutants also exhibit developmental defects and die as larva. Human patients with *de novo* truncating mutations in *WAC* develop Desanto-Shinawi syndrome (OMIM #616708, Hamdan et al., 2014; DeSanto et al., 2015), which is characterized by developmental delay, intellectual disability, dysmorphic features, and hypotonia. In addition, an independent gene knockdown study in *Drosophila* revealed that reduction of Wcy in the nervous system led to habituation defects, a behavior in flies that are linked to cognitive dysfunction (Lugtenberg et al., 2016). Although it is not clear whether defects in mTOR regulation and autophagy are mediating these neurological phenotypes in flies and humans, further characterization of the function of *wcy* in *Drosophila* will likely facilitate the study of *WAC* in the human brain.

### Nrd1 and Ogdh

As previously discussed, mitochondrial dysfunction is often associated with reduced ATP and/or increased ROS that leads to neurodegeneration. By studying mutations in a previously uncharacterized gene that is orthologous to human *Nardilysin convertase* (*NRDC*), we discovered that mitochondrial defects can also lead to neurodegeneration through modulation of mTOR and autophagy (Yoon et al., 2017). *NRDC* encodes a putative metalloprotease whose function and intracellular localization had been debated (Hospital et al., 2000; Ma et al., 2004; Hiraoka et al., 2014). In flies, mutations in *Nardilysin 1 (Nrd1)* exhibit slow, progressive neurodegenerative phenotypes in the visual system. While examining the sequence of *Nrd1*, Yoon et al. (2017) realized that *Drosophila Nrd1* and its orthologous genes all possessed mitochondrial targeting signals at their amino terminus and demonstrated that endogenous Nrd1 is indeed primarily located within the mitochondria *in vivo*. Interestingly, *Nrd1* mutants did not exhibit major defects in ATP synthesis, ETC activity, ROS synthesis or mtDNA levels, suggesting that the mechanism of degeneration is distinct from other mitochondrial related genes. Through metabolomics and proteomics studies followed by targeted biochemical experiments, we found that Nrd1 functions as a mitochondrial molecular chaperone of a Krebs cycle enzyme Oxoglutarate dehydrogenase (Ogdh). Similar to the loss of *Nrd1*, reduction of *Ogdh* via RNAi also exhibited a slow progressive neurodegeneration phenotype. Since Ogdh is a Krebs cycle enzyme that converts α-ketoglutarate (also known as oxoglutarate) to succinyl-CoA, loss of *Ogdh* function leads to a massive accumulation of the metabolite α-ketoglutarate. High-levels of α-ketoglutarate leads to activation of mTORC1 (Chin et al., 2014), resulting in inhibition in autophagy. In fact, the neurodegenerative phenotypes caused by *Nrd1* and *Ogdh* mutations can be suppressed by either reducing the mTORC1 activity via pharmacological administration of rapamycin or by promoting autophagy through overexpression of Atg1 (Yoon et al., 2017).

Although human orthologs of *Nrd1* (*NRDC*) and *Ogdh* (*OGDH* and *OGDHL*) had not been linked to any genetic diseases, we reasoned that mutations in these genes might cause a neurodegenerative disease as we have seen in *Drosophila*. In collaboration with the Baylor-Hopkins Centers for Mendelian Genomics^8^ (Bamshad et al., 2012) and through matchmaking searches using GeneMatcher^9^ (Sobreira et al., 2015), we were able to identify two patients with rare homozygous mutations in *NRDC* and *OGDHL*. The *NRDC* variant was a truncating allele (p.M636VfsX2), similar to the alleles Yoon et al. (2017) isolated from the X-screen (all four *Nrd1* alleles were truncating alleles). The *OGDHL* variant was a missense allele that affects an amino acid that is highly conserved throughout evolution (p.S778L). To determine whether this variant has functional consequences, we ‘humanized’ the Drosophila *Ogdh* gene using recently established T2A-GAL4 methodology (Bellen and Yamamoto, 2015). In this experiment, we expressed the human ortholog with reference or variant sequences in the same spatial and temporal fashion as the fly gene in an *Odgh* mutant background. While the reference transgene was able to rescue the lethality and neurodegenerative phenotypes of the *Ogdh* LOF flies, the variant failed to do so. These results suggested that the p.S778L variant is a LOF mutation and likely contributes to the phenotypes seen in the patient of interest. Both patients exhibited severe developmental delay and presented with slow progressive loss of neurological functions (Yoon et al., 2017), consistent with phenotypes in *Drosophila*. These features were also similar to what had been observed in *Nrd1* knockout mice (Ohno et al., 2009), indicating that these genes play a neuroprotective role in multiple species. Importantly, the progression of neurodegeneration seen upon loss of *Nrd1* and *Ogdh* function is significantly slower than other mitochondrial genes we examined (Jaiswal et al., 2015; Liu et al., 2015). Hence, in contrast to rapid neurodegeneration seen in mutants with high ROS, low ATP or iron accumulation, inhibition of autophagy may cause neurodegeneration through gradual pile-up of toxic materials or chronic wasting of the neurons due to metabolic defects. Identification of more patients with functional mutations in *NRDC* or *OGDHL* will be essential to define the spectrum of phenotypes that are caused by LOF of these genes in humans. In parallel, efforts to explore the links between mitochondrial dysfunction, mTOR signaling, and autophagy will facilitate the mechanistic understanding of these disorders. Such study will likely provide a list of additional genes that may cause related diseases in humans when mutated and further facilitate collaborative research among clinicians, human geneticists and *Drosophila* researchers. Such bidirectional approaches using model organism data to prioritize variants of unknown significance that are found in clinical settings have been successful in identifying new human disease genes (Yamamoto et al., 2014; Harel et al., 2016b; Jakobsdottir et al., 2016; Chao et al., 2017; Liu et al., 2018; Marcogliese et al., 2018; Oláhová et al., 2018; Tan et al., 2018), and are likely to expand as WES and WGS become routine tests in medical examinations for disease diagnosis (Manolio et al., 2017; Wangler et al., 2017).

## Conclusion and Future Directions

*Drosophila* has become an invaluable tool to study the molecular mechanisms of neurodegeneration over the past ∼45 years. Using a forward genetic screening approach, the X-screen discussed here permitted the identification of a number of essential genes required for neuronal maintenance in flies and highlighted several key themes in neurodegeneration including mitochondrial function, protein trafficking, metabolism, and autophagy. Many parallels can be drawn between *Drosophila* and human phenotypes, suggesting that most mechanisms underlying demise of neurons are evolutionarily conserved. The unbiased and non-hypothesis driven phenotype-centric approach described here allowed us to identify unanticipated links between certain genes and cellular mechanisms. For instance, we found that mutations in Krebs cycle enzymes, *kdn* and *Ogdh*, cause neurodegeneration through completely different mechanisms (endolysosomal stress caused by reduced ATP levels disrupting Rh1 trafficking versus inhibition of autophagy due to hyperactivation of mTOR signaling). More recent work using mutants from the X-screen revealed that mutations in *idh3a* [*isocitrate dehydrogenase 3a*, also known as *l(1)G0156*], another Krebs cycle enzyme, exhibited defects in synaptic transmission due to reduction in α-ketoglutarate levels, a phenotype that is not seen in other Krebs cycle mutants (Ugur et al., 2017). These studies highlight the importance of focusing on the phenotypes observed in mutant animals, rather than reported biochemical functions related to the genes of interest. Indeed, knowledge that has been accumulated through *in vitro* studies and cell culture experiments by researchers around the world provide valuable reference points. However, it is critical to perform functional studies in an unbiased fashion *in vivo* since genes and proteins often have unanticipated functions that are only uncovered when assessed in whole organisms. In addition to highlighting the findings in *Drosophila*, we referred to several translational studies performed in mice and humans, one of which led to the identification of new human disease entities (*NRDC* and *OGDHL*). Considering the X-chromosome is ∼15% of the fly genome, similar forward screens on autosomes could greatly increase our understanding of essential genes involved in neuronal maintenance and shed light onto evolutionarily conserved pathways and mechanisms underlying neurodegeneration.

## Author Contributions

All authors listed have made a substantial, direct and intellectual contribution to the work, and approved it for publication.

## Conflict of Interest Statement

The authors declare that the research was conducted in the absence of any commercial or financial relationships that could be construed as a potential conflict of interest.

## Footnotes

1 http://flybase.org/

2 http://flypush.imgen.bcm.tmc.edu/bellenxscreendata/mutantsandphenotypes.xlsx

3 https://bdsc.indiana.edu/

4 http://www.dgrc.kit.ac.jp/

5 https://bdsc.indiana.edu/stocks/chemically_induced_mutations/xlethals.html

6 https://kyotofly.kit.jp/stocks/documents/EMS_X_lethals.html

7 https://www.omim.org/

8 http://mendelian.org/

9 https://genematcher.org/

## References

[B1] Alazami A. M. Patel N. Shamseldin H. E. Anazi S. Al-Dosari M. S. Alzahrani F. (2015). Accelerating novel candidate gene discovery in neurogenetic disorders via whole-exome sequencing of prescreened multiplex consanguineous families. *Cell Rep.* 10 148–161. 10.1016/j.celrep.2014.12.015 25558065

[B2] Alkuraya F. S. (2016). Discovery of mutations for mendelian disorders. *Hum. Genet.* 135 615–623. 10.1007/s00439-016-1664-8 27068822

[B3] Al-Mahdawi S. Pinto R. M. Varshney D. Lawrence L. Lowrie M. B. Hughes S. (2006). GAA repeat expansion mutation mouse models of friedreich ataxia exhibit oxidative stress leading to progressive neuronal and cardiac pathology. *Genomics* 88 580–590. 10.1016/j.ygeno.2006.06.015 16919418 PMC2842930

[B4] Alvarez C. E. (2008). On the origins of arrestin and rhodopsin. *BMC Evol. Biol.* 8:222. 10.1186/1471-2148-8-222 18664266 PMC2515105

[B5] Anderson D. Brenner S. (2008). Seymour benzer (1921–2007). *Nature* 451 139–139. 10.1038/451139a 18185579

[B6] Anderson P. R. Kirby K. Orr W. C. Hilliker A. J. Phillips J. P. (2008). Hydrogen peroxide scavenging rescues frataxin deficiency in a drosophila model of friedreich’s ataxia. *Proc. Natl. Acad. Sci. U.S.A.* 105 611–616. 10.1073/pnas.0709691105 18184803 PMC2206584

[B7] Arber C. E. Li A. Houlden H. Wray S. (2016). Review: insights into molecular mechanisms of disease in neurodegeneration with brain iron accumulation: unifying theories. *Neuropathol. Appl. Neurobiol.* 42 220–241. 10.1111/nan.12242 25870938 PMC4832581

[B8] Aylward E. H. Sparks B. F. Field K. M. Yallapragada V. Shpritz B. D. Rosenblatt A. (2004). Onset and rate of striatal atrophy in preclinical huntington disease. *Neurology* 63 66–72. 10.1212/01.WNL.0000132965.14653.D1 15249612

[B9] Bailey A. P. Koster G. Guillermier C. Hirst E. M. A. MacRae J. I. Lechene C. P. (2015). Antioxidant role for lipid droplets in a stem cell niche of *Drosophila*. *Cell* 163 340–353. 10.1016/j.cell.2015.09.020 26451484 PMC4601084

[B10] Baker E. K. Colley N. J. Zuker C. S. (1994). The cyclophilin homolog ninaa functions as a chaperone, forming a stable complex *in vivo* with its protein target rhodopsin. *EMBO J.* 13 4886–4895. 10.1002/j.1460-2075.1994.tb06816.x 7957056 PMC395429

[B11] Bamshad M. J. Shendure J. A. Valle D. Hamosh A. Lupski J. R. Gibbs R. A. (2012). The centers for mendelian genomics: a new large-scale initiative to identify the genes underlying rare mendelian conditions. *Am. J. Med. Genet. Part A* 158A, 1523–1525. 10.1002/ajmg.a.35470 22628075 PMC3702263

[B12] Batista-Nascimento L. Pimentel C. Andrade Menezes R. Rodrigues-Pousada C. (2012). Iron and neurodegeneration: from cellular homeostasis to disease. *Oxid. Med. Cell. Longev.* 2012 1–8. 10.1155/2012/128647 22701145 PMC3369498

[B13] Bayat V. Thiffault I. Jaiswal M. Tétreault M. Donti T. Sasarman F. (2012). Mutations in the mitochondrial methionyl-trna synthetase cause a neurodegenerative phenotype in flies and a recessive ataxia (ARSAL) in humans. *PLoS Biol.* 10:e1001288. 10.1371/journal.pbio.1001288 22448145 PMC3308940

[B14] Beard J. L. Connor J. R. (2003). Iron status and neural functioning. *Annu. Rev. Nutr.* 23 41–58. 10.1146/annurev.nutr.23.020102.075739 12704220

[B15] Beglopoulos V. Sun X. Saura C. A. Lemere C. A. Kim R. D. Shen J. (2004). Reduced β-amyloid production and increased inflammatory responses in presenilin conditional knock-out mice. *J. Biol. Chem.* 279 46907–46914. 10.1074/jbc.M409544200 15345711

[B16] Bellen H. J. Yamamoto S. (2015). Morgan’s legacy: fruit flies and the functional annotation of conserved genes. *Cell* 163 12–14. 10.1016/j.cell.2015.09.009 26406362 PMC4783153

[B17] Berger J. Senti K.-A. Senti G. Newsome T. P. Åsling B. Dickson B. J. (2008). Systematic identification of genes that regulate neuronal wiring in the *Drosophila* visual system. *PLoS Genet.* 4:e1000085. 10.1371/journal.pgen.1000085 18516287 PMC2377342

[B18] Biasiotto G. Lorenzo D. Di Archetti S. Zanella I. (2016). Iron and neurodegeneration: is ferritinophagy the link? *Mol. Neurobiol.* 53 5542–5574. 10.1007/s12035-015-9473-y 26468157

[B19] Bier E. (2005). Drosophila, the golden bug, emerges as a tool for human genetics. *Nat. Rev. Genet.* 6 9–23. 10.1038/nrg1503 15630418

[B20] Bilen J. Bonini N. M. (2005). *Drosophila* as a model for human neurodegenerative disease. *Annu. Rev. Genet.* 39 153–171. 10.1146/annurev.genet.39.110304.095804 16285856

[B21] Blumenstiel J. P. Noll A. C. Griffiths J. A. Perera A. G. Walton K. N. Gilliland W. D. (2009). Identification of EMS-induced mutations in *Drosophila Melanogaster* by whole-genome sequencing. *Genetics* 182 25–32. 10.1534/genetics.109.101998 19307605 PMC2674820

[B22] Blumkin L. Michelson M. Leshinsky-Silver E. Kivity S. Lev D. Lerman-Sagie T. (2010). Congenital ataxia, mental retardation, and dyskinesia associated with a novel cacna1a mutation. *J. Child Neurol.* 25 892–897. 10.1177/0883073809351316 20097664

[B23] Boycott K. M. Rath A. Chong J. X. Hartley T. Alkuraya F. S. Baynam G. (2017). International cooperation to enable the diagnosis of all rare genetic diseases. *Am. J. Hum. Genet.* 100 695–705. 10.1016/j.ajhg.2017.04.003 28475856 PMC5420351

[B24] Brand A. H. Perrimon N. (1993). Targeted gene expression as a means of altering cell fates and generating dominant phenotypes. *Development* 118 401–415. 8223268 10.1242/dev.118.2.401

[B25] Brand M. D. Nicholls D. G. (2011). Assessing mitochondrial dysfunction in cells. *Biochem. J.* 435 297–312. 10.1042/BJ20110162 21726199 PMC3076726

[B26] Bratic A. Wredenberg A. Grönke S. Stewart J. B. Mourier A. Ruzzenente B. (2011). The bicoid stability factor controls polyadenylation and expression of specific mitochondrial MRNAs in *Drosophila Melanogaster*. *PLoS Genet.* 7:e1002324. 10.1371/journal.pgen.1002324 22022283 PMC3192837

[B27] Brown G. K. Otero L. J. LeGris M. Brown R. M. (1994). Pyruvate dehydrogenase deficiency. *J. Med. Genet.* 31 875–879. 10.1136/jmg.31.11.875 7853374 PMC1016663

[B28] Buchanan R. L. Benzer S. (1993). Defective glia in the drosophila brain degeneration mutant drop-dead. *Neuron* 10 839–850. 10.1016/0896-6273(93)90200-B 8494644

[B29] Burman J. L. Itsara L. S. Kayser E.-B. Suthammarak W. Wang A. M. Kaeberlein M. (2014). A Drosophila model of mitochondrial disease caused by a complex i mutation that uncouples proton pumping from electron transfer. *Dis. Model. Mech.* 7 1165–1174. 10.1242/dmm.015321 25085991 PMC4174527

[B30] Campuzano V. Montermini L. Moltò M. D. Pianese L. Cossée M. Cavalcanti F. (1996). Friedreich’s ataxia: autosomal recessive disease caused by an intronic GAA triplet repeat expansion. *Science* 271 1423–1427. 10.1126/science.271.5254.1423 8596916

[B31] Chan D. C. (2012). Fusion and fission: interlinked processes critical for mitochondrial health. *Annu. Rev. Genet.* 46 265–287. 10.1146/annurev-genet-110410-132529 22934639

[B32] Chao H.-T. Davids M. Burke E. Pappas J. G. Rosenfeld J. A. McCarty A. J. (2017). A syndromic neurodevelopmental disorder caused by de novo variants in EBF3. *Am. J. Hum. Genet.* 100 128–137. 10.1016/j.ajhg.2016.11.018 28017372 PMC5223093

[B33] Charng W.-L. Yamamoto S. Bellen H. J. (2014). Shared mechanisms between drosophila peripheral nervous system development and human neurodegenerative diseases. *Curr. Opin. Neurobiol.* 27 158–164. 10.1016/j.conb.2014.03.001 24762652 PMC4122633

[B34] Chen K. Ho T. S.-Y. Lin G. Tan K. L. Rasband M. N. Bellen H. J. (2016a). Loss of frataxin activates the iron/sphingolipid/PDK1/Mef2 pathway in mammals. *eLife* 5:e20732. 10.7554/eLife.20732 27901468 PMC5130293

[B35] Chen K. Lin G. Haelterman N. A. Ho T. S.-Y. Li T. Li Z. (2016b). Loss of frataxin induces iron toxicity, sphingolipid synthesis, and Pdk1/Mef2 activation, leading to neurodegeneration. *eLife* 5:e16043. 10.7554/eLife.16043 27343351 PMC4956409

[B36] Chen L. Feany M. B. (2005). α-synuclein phosphorylation controls neurotoxicity and inclusion formation in a drosophila model of parkinson disease. *Nat. Neurosci.* 8 657–663. 10.1038/nn1443 15834418

[B37] Chin R. M. Fu X. Pai M. Y. Vergnes L. Hwang H. Deng G. (2014). The metabolite α-ketoglutarate extends lifespan by inhibiting ATP synthase and TOR. *Nature* 510 397–401. 10.1038/nature13264 24828042 PMC4263271

[B38] Chinchore Y. Mitra A. Dolph P. J. (2009). Accumulation of rhodopsin in late endosomes triggers photoreceptor cell degeneration. *PLoS Genet.* 5:e1000377. 10.1371/journal.pgen.1000377 19214218 PMC2633617

[B39] Chong J. X. Buckingham K. J. Jhangiani S. N. Boehm C. Sobreira N. Smith J. D. (2015). The genetic basis of mendelian phenotypes: discoveries, challenges, and opportunities. *Am. J. Hum. Genet.* 97 199–215. 10.1016/j.ajhg.2015.06.009 26166479 PMC4573249

[B40] Chung K. W. Kim S. B. Park K. D. Choi K. G. Lee J. H. Eun H. W. (2006). Early onset severe and late-onset mild charcot-marie-tooth disease with mitofusin 2 (MFN2) mutations. *Brain* 129 2103–2118. 10.1093/brain/awl174 16835246

[B41] Cipolat S. Brito O. M. de Dal Zilio B. Scorrano L. (2004). OPA1 requires mitofusin 1 to promote mitochondrial fusion. *Proc. Natl. Acad. Sci. U.S.A.* 101 15927–15932. 10.1073/pnas.0407043101 15509649 PMC528769

[B42] Cook M. S. Cazin C. Amoyel M. Yamamoto S. Bach E. Nystul T. (2017). Neutral competition for *Drosophila* follicle and cyst stem cell niches requires vesicle trafficking genes. *Genetics* 206 1417–1428. 10.1534/genetics.117.201202 28512187 PMC5500140

[B43] Cook R. K. Christensen S. J. Deal J. A. Coburn R. A. Deal M. E. Gresens J. M. (2012). The generation of chromosomal deletions to provide extensive coverage and subdivision of the *Drosophila melanogaster* genome. *Genome Biol.* 13:R21. 10.1186/gb-2012-13-3-r21 22445104 PMC3439972

[B44] Cook R. K. Deal M. E. Deal J. A. Garton R. D. Brown C. A. Ward M. E. (2010). A new resource for characterizing x-linked genes in *Drosophila melanogaster*: systematic coverage and subdivision of the x chromosome with nested, y-linked duplications. *Genetics* 186 1095–1109. 10.1534/genetics.110.123265 20876560 PMC2998296

[B45] Dahl H.-H. M. (1998). Invited editorial getting to the nucleus of mitochondrial disorders: identification of respiratory chain-enzyme genes causing leigh syndrome. *Am. J. Hum. Genet* 63 1594–1597. 9837811 10.1086/302169 PMC1377630

[B46] Da-Rè C. Stockum S. von Biscontin A. Millino C. Cisotto P. Zordan M. A. (2014). Leigh syndrome in *Drosophila melanogaster*. *J. Biol. Chem.* 289 29235–29246. 10.1074/jbc.M114.602938 25164807 PMC4200275

[B47] David-Morrison G. Xu Z. Rui Y.-N. Charng W.-L. Jaiswal M. Yamamoto S. (2016). WAC regulates MTOR activity by acting as an adaptor for the TTT and pontin/reptin complexes. *Dev. Cell* 36 139–151. 10.1016/j.devcel.2015.12.019 26812014 PMC4730548

[B48] de Brito O. M. Scorrano L. (2008). Mitofusin 2 tethers endoplasmic reticulum to mitochondria. *Nature* 456 605–610. 10.1038/nature07534 19052620

[B49] Debattisti V. Scorrano L. (2013). *D. melanogaster*, mitochondria and neurodegeneration: small model organism, big discoveries. *Mol. Cell. Neurosci.* 55 77–86. 10.1016/j.mcn.2012.08.007 22940086

[B50] Delettre C. Lenaers G. Griffoin J. M. Gigarel N. Lorenzo C. Belenguer P. (2000). Nuclear gene OPA1, encoding a mitochondrial dynamin-related protein, is mutated in dominant optic atrophy. *Nat. Genet.* 26 207–210. 10.1038/79936 11017079

[B51] den Hollander A. I. ten Brink J. B. de Kok Y. J. van Soest S. van den Born L. I. van Driel M. A. (1999). Mutations in a human homologue of *Drosophila* crumbs cause retinitis pigmentosa (RP12). *Nat. Genet* 23 217–221. 10.1038/13848 10508521

[B52] Denier C. Ducros A. Durr A. Eymard B. Chassande B. Tournier-Lasserve E. (2001). Missense CACNA1A mutation causing episodic ataxia type 2. *Arch. Neurol.* 58 292–295. 10.1001/archneur.58.2.292 11176968

[B53] DeSanto C. D’Aco K. Araujo G. C. Shannon N. Study D. Vernon H. (2015). WAC loss-of-function mutations cause a recognisable syndrome characterised by dysmorphic features, developmental delay and hypotonia and recapitulate 10p11.23 microdeletion syndrome. *J. Med. Genet.* 52 754–761. 10.1136/jmedgenet-2015-103069 26264232

[B54] Dickman D. K. Kurshan P. T. Schwarz T. L. (2008). Mutations in a *Drosophila* 2 voltage-gated calcium channel subunit reveal a crucial synaptic function. *J. Neurosci.* 28 31–38. 10.1523/JNEUROSCI.4498-07.2008 18171920 PMC6671140

[B55] Dinter E. Saridaki T. Nippold M. Plum S. Diederichs L. Komnig D. (2016). Rab7 induces clearance of α-synuclein aggregates. *J. Neurochem.* 138 758–774. 10.1111/jnc.13712 27333324

[B56] Dobrosotskaya I. Y. Seegmiller A. C. Brown M. S. Goldstein J. L. Rawson R. B. (2002). Regulation of SREBP processing and membrane lipid production by phospholipids in *Drosophila*. *Science* 296 879–883. 10.1126/science.1071124 11988566

[B57] Dryja T. P. McGee T. L. Reichel E. Hahn L. B. Cowley G. S. Yandell D. W. (1990). A point mutation of the rhodopsin gene in one form of retinitis pigmentosa. *Nature* 343 364–366. 10.1038/343364a0 2137202

[B58] Ducros A. Denier C. Joutel A. Cecillon M. Lescoat C. Vahedi K. (2001). The clinical spectrum of familial hemiplegic migraine associated with mutations in a neuronal calcium channel. *N. Engl. J. Med.* 345 17–24. 10.1056/NEJM200107053450103 11439943

[B59] Duffy J. B. Harrison D. A. Perrimon N. (1998). Identifying loci required for follicular patterning using directed mosaics. *Development* 125 2263–2271. 9584125 10.1242/dev.125.12.2263

[B60] Dusek P. Schneider S. A. (2012). Neurodegeneration with brain iron accumulation. *Curr. Opin. Neurol.* 25 499–506. 10.1097/WCO.0b013e3283550cac 22691760

[B61] Edwards T. N. Nuschke A. C. Nern A. Meinertzhagen I. A. (2012). Organization and metamorphosis of glia in the *Drosophila* visual system. *J. Comp. Neurol.* 520 2067–2085. 10.1002/cne.23071 22351615

[B62] EPI4K Consortium Epilepsy Phenome Genome Project Allen A. S. Berkovic S. F. Cossette P. Delanty N. (2013). De novo mutations in epileptic encephalopathies. *Nature* 501 217–221. 10.1038/nature12439 23934111 PMC3773011

[B63] Felix R. (2002). Insights from mouse models of absence epilepsy into Ca2+ channel physiology and disease etiology. *Cell. Mol. Neurobiol.* 22 103–120. 10.1023/A:1019807719343 12363194 PMC11533750

[B64] Feng R. Wang H. Wang J. Shrom D. Zeng X. Tsien J. Z. (2004). Forebrain degeneration and ventricle enlargement caused by double knockout of alzheimer’s presenilin-1 and presenilin-2. *Proc. Natl. Acad. Sci. U.S.A.* 101 8162–8167. 10.1073/pnas.0402733101 15148382 PMC419574

[B65] Fouquet W. Owald D. Wichmann C. Mertel S. Depner H. Dyba M. (2009). Maturation of active zone assembly by *Drosophila* bruchpilot. *J. Cell Biol.* 186 129–145. 10.1083/jcb.200812150 19596851 PMC2712991

[B66] Frake R. A. Ricketts T. Menzies F. M. Rubinsztein D. C. (2015). Autophagy and neurodegeneration. *J. Clin. Invest.* 125 65–74. 10.1172/JCI73944 25654552 PMC4382230

[B67] Fritz K. S. Petersen D. R. (2013). An overview of the chemistry and biology of reactive aldehydes. *Free Radic. Biol. Med.* 59 85–91. 10.1016/j.freeradbiomed.2012.06.025 22750507 PMC3540155

[B68] Frost B. Hemberg M. Lewis J. Feany M. B. (2014). Tau promotes neurodegeneration through global chromatin relaxation. *Nat. Neurosci.* 17 357–366. 10.1038/nn.3639 24464041 PMC4012297

[B69] Galy A. Roux M. J. Sahel J. A. Léveillard T. Giangrande A. (2005). Rhodopsin maturation defects induce photoreceptor death by apoptosis: a fly model for rhodopsinpro23his human retinitis pigmentosa. *Hum. Mol. Genet.* 14 2547–2557. 10.1093/hmg/ddi258 16049034

[B70] Gambis A. Dourlen P. Steller H. Mollereau B. (2011). Two-color *in vivo* imaging of photoreceptor apoptosis and development in *Drosophila*. *Dev. Biol.* 351 128–134. 10.1016/j.ydbio.2010.12.040 21215264 PMC3051417

[B71] Gerber S. Charif M. Chevrollier A. Chaumette T. Angebault C. Kane M. S. (2017). Mutations in DNM1L, as in OPA1, result in dominant optic atrophy despite opposite effects on mitochondrial fusion and fission. *Brain* 140 2586–2596. 10.1093/brain/awx219 28969390

[B72] Giguère N. Burke Nanni S. Trudeau L.-E. (2018). On cell loss and selective vulnerability of neuronal populations in parkinson’s disease. *Front. Neurol.* 9:455 10.3389/fneur.2018.00455 PMC6018545 29971039

[B73] Golic K. G. Lindquist S. (1989). The FLP recombinase of yeast catalyzes site-specific recombination in the *Drosophila* genome. *Cell* 59 499–509. 10.1016/0092-8674(89)90033-0 2509077

[B74] Gonzalez M. Booven D. Van Hulme W. Ulloa R. Lebrigio R. Osterloh J. (2012). Whole genome sequencing and a new bioinformatics platform allow for rapid gene identification in *D. melanogaster* EMS screens. *Biology* 1 766–777. 10.3390/biology1030766 24832518 PMC4009818

[B75] Gramates L. S. Marygold S. J. Santos G. dos Urbano J.-M. Antonazzo G. Matthews B. B. (2017). Flybase at 25: looking to the future. *Nucleic Acids Res.* 45 D663–D671. 10.1093/nar/gkw1016 27799470 PMC5210523

[B76] Greeve I. Kretzschmar D. Tschäpe J.-A. Beyn A. Brellinger C. Schweizer M. (2004). Age-dependent neurodegeneration and alzheimer-amyloid plaque formation in transgenic *Drosophila*. *J. Neurosci.* 24 3899–3906. 10.1523/JNEUROSCI.0283-04.2004 15102905 PMC6729409

[B77] Gu H. Zou Y. R. Rajewsky K. (1993). Independent control of immunoglobulin switch recombination at individual switch regions evidenced through cre-loxp-mediated gene targeting. *Cell* 73 1155–1164. 10.1016/0092-8674(93)90644-6 8513499

[B78] Gueneau L. Fish R. J. Shamseldin H. E. Voisin N. Tran Mau-Them F. Preiksaitiene E. (2018). KIAA1109 variants are associated with a severe disorder of brain development and arthrogryposis. *Am. J. Hum. Genet.* 102 116–132. 10.1016/j.ajhg.2017.12.002 29290337 PMC5777449

[B79] Guo F. Liu X. Cai H. Le W. (2018). Autophagy in neurodegenerative diseases: pathogenesis and therapy. *Brain Pathol.* 28 3–13. 10.1111/bpa.12545 28703923 PMC5739982

[B80] Guo Y. Livne-Bar I. Zhou L. Boulianne G. L. (1999). Drosophila presenilin is required for neuronal differentiation and affects notch subcellular localization and signaling. *J. Neurosci.* 19 8435–8442. 10.1523/JNEUROSCI.19-19-08435.1999 10493744 PMC6783002

[B81] Haelterman N. A. Jiang L. Li Y. Bayat V. Sandoval H. Ugur B. (2014a). Large-scale identification of chemically induced mutations in *Drosophila melanogaster*. *Genome Res.* 24 1707–1718. 10.1101/gr.174615.114 25258387 PMC4199363

[B82] Haelterman N. A. Yoon W. H. Sandoval H. Jaiswal M. Shulman J. M. Bellen H. J. (2014b). A mitocentric view of parkinson’s disease. *Annu. Rev. Neurosci.* 37 137–159. 10.1146/annurev-neuro-071013-014317 24821430 PMC4659514

[B83] Haft C. R. De la Luz Sierra M. de Bafford R. Lesniak M. A. Barr V. A. Taylor I. (2000). Human orthologs of yeast vacuolar protein sorting proteins Vps26, 29, and 35: assembly into multimeric complexes. *Mol. Biol. Cell* 11 4105–4116. 10.1091/mbc.11.12.4105 11102511 PMC15060

[B84] Hamdan F. F. Srour M. Capo-Chichi J.-M. Daoud H. Nassif C. Patry L. (2014). De novo mutations in moderate or severe intellectual disability. *PLoS Genet.* 10:e1004772. 10.1371/journal.pgen.1004772 25356899 PMC4214635

[B85] Harel T. Yesil G. Bayram Y. Coban-Akdemir Z. Charng W.-L. Karaca E. (2016a). Monoallelic and biallelic variants in emc1 identified in individuals with global developmental delay, hypotonia, scoliosis, and cerebellar atrophy. *Am. J. Hum. Genet.* 98 562–570. 10.1016/j.ajhg.2016.01.011 26942288 PMC4800043

[B86] Harel T. Yoon W. H. Garone C. Gu S. Coban-Akdemir Z. Eldomery M. K. (2016b). Recurrent de novo and biallelic variation of ATAD3A, encoding a mitochondrial membrane protein, results in distinct neurological syndromes. *Am. J. Hum. Genet.* 99 831–845. 10.1016/j.ajhg.2016.08.007 27640307 PMC5065660

[B87] Hayashi G. Cortopassi G. (2015). Oxidative stress in inherited mitochondrial diseases. *Free Radic. Biol. Med.* 88(Pt A), 10–17. 10.1016/j.freeradbiomed.2015.05.039 26073122 PMC4593728

[B88] Hiraoka Y. Matsuoka T. Ohno M. Nakamura K. Saijo S. Matsumura S. (2014). Critical roles of nardilysin in the maintenance of body temperature homoeostasis. *Nat. Commun.* 5:3224. 10.1038/ncomms4224 24492630 PMC3926010

[B89] Hospital V. Chesneau V. Balogh A. Joulie C. Seidah N. G. Cohen P. (2000). N-arginine dibasic convertase (Nardilysin) isoforms are soluble dibasic-specific metalloendopeptidases that localize in the cytoplasm and at the cell surface. *Biochem. J.* 349(Pt 2), 587–597. 10.1042/bj3490587 10880358 PMC1221182

[B90] Hotta Y. Benzer S. (1972). Mapping of behaviour in *Drosophila mosaics*. *Nature* 240 527–535. 10.1038/240527a0 4568399

[B91] Hou J. Tamura T. Kidokoro Y. (2008). Delayed synaptic transmission in *Drosophila cacophony null* embryos. *J. Neurophysiol.* 100 2833–2842. 10.1152/jn.90342.2008 18815348

[B92] Issop L. Rone M. B. Papadopoulos V. (2013). Organelle plasticity and interactions in cholesterol transport and steroid biosynthesis. *Mol. Cell. Endocrinol.* 371 34–46. 10.1016/j.mce.2012.12.003 23246788

[B93] Jafar-Nejad H. Andrews H. K. Acar M. Bayat V. Wirtz-Peitz F. Mehta S. Q. (2005). Sec15, a component of the exocyst, promotes notch signaling during the asymmetric division of *Drosophila* sensory organ precursors. *Dev. Cell* 9 351–363. 10.1016/j.devcel.2005.06.010 16137928

[B94] Jaiswal M. Haelterman N. A. Sandoval H. Xiong B. Donti T. Kalsotra A. (2015). Impaired mitochondrial energy production causes light-induced photoreceptor degeneration independent of oxidative stress. *PLoS Biol.* 13:e1002197. 10.1371/journal.pbio.1002197 26176594 PMC4503542

[B95] Jaiswal M. Sandoval H. Zhang K. Bayat V. Bellen H. J. (2012). Probing mechanisms that underlie human neurodegenerative diseases in *Drosophila*. *Annu*. *Rev. Genet* 46 371–396. 10.1146/annurev-genet-110711-155456 22974305 PMC3663445

[B96] Jakobsdottir J. Lee S. J. van der Bis J. C. Chouraki V. Li-Kroeger D. Yamamoto S. (2016). Rare functional variant in TM2D3 is associated with late-ons etalzheimer’s disease. *PLoS Genet* 12:e1006327. 10.1371/journal.pgen.1006327 27764101 PMC5072721

[B97] James B. D. Leurgans S. E. Hebert L. E. Scherr P. A. Yaffe K. Bennett D. A. (2014). Contribution of alzheimer disease to mortality in the United States. *Neurology* 82 1045–1050. 10.1212/WNL.0000000000000240 24598707 PMC3962992

[B98] Jimenez-Sanchez M. Lam W. Hannus M. Sönnichsen B. Imarisio S. Fleming A. (2015). SiRNA screen identifies QPCT as a druggable target for huntington’s disease. *Nat. Chem. Biol.* 11 347–354. 10.1038/nchembio.1790 25848931 PMC4696152

[B99] Jun K. Piedras-Rentería E. S. Smith S. M. Wheeler D. B. Lee S. B. Lee T. G. (1999). Ablation of P/Q-type Ca(2+) channel currents, altered synaptic transmission, and progressive ataxia in mice lacking the Alpha(1A)-subunit. *Proc. Natl. Acad. Sci. U.S.A.* 96 15245–15250. 10.1073/pnas.96.26.15245 10611370 PMC24805

[B100] Kang J. Shin S. Perrimon N. Shen J. (2017). An evolutionarily conserved role of presenilin in neuronal protection in the aging *Drosophila* brain. *Genetics* 206 1479–1493. 10.1534/genetics.116.196881 28495961 PMC5500145

[B101] Kawamata H. Manfredi G. (2017). Proteinopathies and OXPHOS dysfunction in neurodegenerative diseases. *J. Cell Biol.* 216 3917–3929. 10.1083/jcb.201709172 29167179 PMC5716291

[B102] Kim J. Basak J. M. Holtzman D. M. (2009). The role of apolipoprotein E in alzheimer’s disease. *Neuron* 63 287–303. 10.1016/j.neuron.2009.06.026 19679070 PMC3044446

[B103] Kim M. Ho A. Lee J. H. (2017). Autophagy and human neurodegenerative diseases—a fly’s perspective. *Int. J. Mol. Sci.* 18:1596. 10.3390/ijms18071596 28737703 PMC5536083

[B104] Kiselev A. Subramaniam S. (1994). Activation and regeneration of rhodopsin in the insect visual cycle. *Science* 266 1369–1373. 10.1126/science.7973725 7973725

[B105] Kohda M. Tokuzawa Y. Kishita Y. Nyuzuki H. Moriyama Y. Mizuno Y. (2016). A comprehensive genomic analysis reveals the genetic landscape of mitochondrial respiratory chain complex deficiencies. *PLoS Genet.* 12:e1005679. 10.1371/journal.pgen.1005679 26741492 PMC4704781

[B106] Konovalova S. Tyynismaa H. (2013). Mitochondrial aminoacyl-TRNA synthetases in human disease. *Mol. Genet. Metab.* 108 206–211. 10.1016/j.ymgme.2013.01.010 23433712

[B107] Koopman W. J. H. Verkaart S. Emst-de Vries S. E. van Grefte S. Smeitink J. A. M. Nijtmans L. G. J. (2008). Mitigation of NADH: ubiquinone oxidoreductase deficiency by chronic trolox treatment. *Biochim. Biophys. Acta Bioenerg* 1777 853–859. 10.1016/j.bbabio.2008.03.028 18435906

[B108] Kretzschmar D. Hasan G. Sharma S. Heisenberg M. Benzer S. (1997). The swiss cheese mutant causes glial hyperwrapping and brain degeneration in *Drosophila*. *J. Neurosci.* 17 7425–7432. 10.1523/JNEUROSCI.17-19-07425.1997 9295388 PMC6573436

[B109] Kruse S. E. Watt W. C. Marcinek D. J. Kapur R. P. Schenkman K. A. Palmiter R. D. (2008). Mice with mitochondrial complex i deficiency develop a fatal encephalomyopathy. *Cell Metab.* 7 312–320. 10.1016/j.cmet.2008.02.004 18396137 PMC2593686

[B110] Kunte A. S. Matthews K. A. Rawson R. B. (2006). Fatty acid auxotrophy in *Drosophila* larvae lacking SREBP. *Cell Metab.* 3 439–448. 10.1016/j.cmet.2006.04.011 16753579

[B111] Lake N. J. Compton A. G. Rahman S. Thorburn D. R. (2016). Leigh syndrome: one disorder, more than 75 monogenic causes. *Ann. Neurol.* 79 190–203. 10.1002/ana.24551 26506407

[B112] Lamers I. J. C. Reijnders M. R. F. Venselaar H. Kraus A. Ddd Study S. Jansen S. (2017). Recurrent de novo mutations disturbing the GTP/GDP binding pocket of RAB11B cause intellectual disability and a distinctive brain phenotype. *Am. J. Hum. Genet.* 101 824–832. 10.1016/j.ajhg.2017.09.015 29106825 PMC5673605

[B113] Lauwers E. Verstreken P. (2018). Assaying mutants of clathrin-mediated endocytosis in the fly eye. *Methods Mol. Biol.* 1847 109–119. 10.1007/978-1-4939-8719-1_9 30129013

[B114] Lee T. Winter C. Marticke S. S. Lee A. Luo L. (2000). Essential roles of *Drosophila* RhoA in the regulation of neuroblast proliferation and dendritic but not axonal morphogenesis. *Neuron* 25 307–316. 10.1016/S0896-6273(00)80896-X 10719887

[B115] Leigh D. (1951). Subacute necrotizing encephalomyelopathy in an infant. *J. Neurol. Neurosurg. Psychiatry* 14 216–221. 10.1136/jnnp.14.3.216 14874135 PMC499520

[B116] Leister D. (2005). Origin, evolution and genetic effects of nuclear insertions of organelle DNA. *Trends Genet.* 21 655–663. 10.1016/j.tig.2005.09.004 16216380

[B117] Lenz S. Karsten P. Schulz J. B. Voigt A. (2013). *Drosophila* as a screening tool to study human neurodegenerative diseases. *J. Neurochem.* 127 453–460. 10.1111/jnc.12446 24028575

[B118] Lessing D. Bonini N. M. (2009). Maintaining the brain: insight into human neurodegeneration from *Drosophila melanogaster* mutants. *Nat. Rev. Genet.* 10 359–370. 10.1038/nrg2563 19434080 PMC2820605

[B119] Letts J. A. Fiedorczuk K. Sazanov L. A. (2016). The architecture of respiratory supercomplexes. *Nature* 537 644–648. 10.1038/nature19774 27654913

[B120] Levine B. Kroemer G. (2008). Autophagy in the pathogenesis of disease. *Cell* 132 27–42. 10.1016/j.cell.2007.12.018 18191218 PMC2696814

[B121] Li Z.-Y. Jacobson S. G. Milam A. H. (1994). Autosomal dominant retinitis pigmentosa caused by the threonine-17-methionine rhodopsin mutation: retinal histopathology and immunocytochemistry. *Exp. Eye Res.* 58 397–408. 10.1006/exer.1994.1032 7925677

[B122] Liao T. S. V. Call G. B. Guptan P. Cespedes A. Marshall J. Yackle K. (2006). An efficient genetic screen in *Drosophila* to identify nuclear-encoded genes with mitochondrial function. *Genetics* 174 525–533. 10.1534/genetics.106.061705 16849596 PMC1569793

[B123] Lin G. Lee P.-T. Chen K. Mao D. Tan K. L. Zuo Z. (2018). Phospholipase PLA2G6, a parkinsonism-associated gene, affects Vps26 and Vps35, retromer function, and ceramide levels, similar to α-synuclein gain. *Cell Metab.* 28 605–618. 10.1016/j.cmet.2018.05.019 29909971

[B124] Lin M. T. Beal M. F. (2006). Mitochondrial dysfunction and oxidative stress in neurodegenerative diseases. *Nature* 443 787–795. 10.1038/nature05292 17051205

[B125] Ling D. Song H.-J. Garza D. Neufeld T. P. Salvaterra P. M. (2009). Abeta42-induced neurodegeneration via an age-dependent autophagic-lysosomal injury in *Drosophila*. *PLoS One* 4:e4201. 10.1371/journal.pone.0004201 19145255 PMC2626277

[B126] Lipton J. O. Sahin M. (2014). The neurology of MTOR. *Neuron* 84 275–291. 10.1016/j.neuron.2014.09.034 25374355 PMC4223653

[B127] Liu L. MacKenzie K. R. Putluri N. Maletiæ-Savatiæ M. Bellen H. J. (2017). The glia-neuron lactate shuttle and elevated ros promote lipid synthesis in neurons and lipid droplet accumulation in glia via APOE/D. *Cell Metab.* 26 719.e–737.e. 10.1016/j.cmet.2017.08.024 28965825 PMC5677551

[B128] Liu L. Zhang K. Sandoval H. Yamamoto S. Jaiswal M. Sanz E. (2015). Glial lipid droplets and ROS induced by mitochondrial defects promote neurodegeneration. *Cell* 160 177–190. 10.1016/j.cell.2014.12.019 25594180 PMC4377295

[B129] Liu N. Schoch K. Luo X. Pena L. D. M. Bhavana V. H. Kukolich M. K. (2018). Functional variants in TBX2 are associated with a syndromic cardiovascular and skeletal developmental disorder. *Hum. Mol. Genet.* 27 2454–2465. 10.1093/hmg/ddy146 29726930 PMC6030957

[B130] Liu W. Tang F.-L. Erion J. Xiao H. Ye J. Xiong W.-C. (2014). Vps35 haploinsufficiency results in degenerative-like deficit in mouse retinal ganglion neurons and impairment of optic nerve injury-induced gliosis. *Mol. Brain* 7:10. 10.1186/1756-6606-7-10 24512632 PMC4016418

[B131] Lugtenberg D. Reijnders M. R. Fenckova M. Bijlsma E. K. Bernier R. van Bon B. W. M. (2016). De novo loss-of-function mutations in WAC cause a recognizable intellectual disability syndrome and learning deficits in *Drosophila*. *Eur. J. Hum. Genet.* 24 1145–1153. 10.1038/ejhg.2015.282 26757981 PMC4970694

[B132] Luo X. Rosenfeld J. A. Yamamoto S. Harel T. Zuo Z. Hall M. (2017). Clinically severe CACNA1A alleles affect synaptic function and neurodegeneration differentially. *PLoS Genet.* 13:e1006905. 10.1371/journal.pgen.1006905 28742085 PMC5557584

[B133] Luschnig S. Moussian B. Krauss J. Desjeux I. Perkovic J. Nüsslein-Volhard C. (2004). An F1 genetic screen for maternal-effect mutations affecting embryonic pattern formation in *Drosophila melanogaster*. *Genetics* 167 325–342. 10.1534/genetics.167.1.325 15166158 PMC1470860

[B134] Lush M. J. Li Y. Read D. J. Willis A. C. Glynn P. (1998). Neuropathy target esterase and a homologous drosophila neurodegeneration-associated mutant protein contain a novel domain conserved from bacteria to man. *Biochem. J.* 332( Pt 1), 1–4. 10.1042/bj3320001 9576844 PMC1219444

[B135] Ly C. V. Yao C.-K. Verstreken P. Ohyama T. Bellen H. J. (2008). Straightjacket is required for the synaptic stabilization of cacophony, a voltage-gated calcium channel α 1 subunit. *J. Cell Biol.* 181 157–170. 10.1083/jcb.200712152 18391075 PMC2287295

[B136] Ma Z. Chow K. M. Yao J. Hersh L. B. (2004). Nuclear shuttling of the peptidase nardilysin. *Arch. Biochem. Biophys.* 422 153–160. 10.1016/j.abb.2003.11.024 14759602

[B137] MacKenzie E. L. Iwasaki K. Tsuji Y. (2008). Intracellular iron transport and storage: from molecular mechanisms to health implications. *Antioxid. Redox Signal.* 10 997–1030. 10.1089/ars.2007.1893 18327971 PMC2932529

[B138] Mancebo R. Zhou X. Shillinglaw W. Henzel W. Macdonald P. M. (2001). BSF binds specifically to the bicoid mrna 3’ untranslated region and contributes to stabilization of bicoid MRNA. *Mol. Cell. Biol.* 21 3462–3471. 10.1128/MCB.21.10.3462-3471.2001 11313472 PMC100268

[B139] Manczak M. Park B. S. Jung Y. Reddy P. H. (2004). Differential expression of oxidative phosphorylation genes in patients with alzheimer’s disease: implications for early mitochondrial dysfunction and oxidative damage. *Neuro Mol. Med.* 5 147–162. 10.1385/NMM:5:2:147 15075441

[B140] Manolio T. A. Fowler D. M. Starita L. M. Haendel M. A. MacArthur D. G. Biesecker L. G. (2017). Bedside back to bench: building bridges between basic and clinical genomic research. *Cell* 169 6–12. 10.1016/j.cell.2017.03.005 28340351 PMC5511379

[B141] Marcogliese P. C. Abuaish S. Kabbach G. Abdel-Messih E. Seang S. Li G. (2017). LRRK2(I2020T) functional genetic interactors that modify eye degeneration and dopaminergic cell loss in *Drosophila*. *Hum. Mol. Genet.* 26 1247–1257. 10.1093/hmg/ddx030 28158614 PMC6075539

[B142] Marcogliese P. C. Shashi V. Spillmann R. C. Stong N. Rosenfeld J. A. Koenig M. K. (2018). IRF2BPL is associated with neurological phenotypes. *Am. J. Hum. Genet.* 103 245–260. 10.1016/j.ajhg.2018.07.006 30057031 PMC6081494

[B143] Martini-Stoica H. Xu Y. Ballabio A. Zheng H. (2016). The autophagy–lysosomal pathway in neurodegeneration: a TFEB perspective. *Trends Neurosci.* 39 221–234. 10.1016/j.tins.2016.02.002 26968346 PMC4928589

[B144] Mato M. Ookawara S. Mashiko T. Sakamoto A. Mato T. K. Maeda N. (1999). Regional difference of lipid distribution in brain of apolipoprotein E deficient mice. *Anat. Rec.* 256 165–176. 10.1002/(SICI)1097-0185(19991001)256:2<165::AID-AR7>3.0.CO;2-Y 10486514

[B145] McGurk L. Berson A. Bonini N. M. (2015). *Drosophila* as an *in vivo* model for human neurodegenerative disease. *Genetics* 201 377–402. 10.1534/genetics.115.179457 26447127 PMC4596656

[B146] McMillan K. J. Gallon M. Jellett A. P. Clairfeuille T. Tilley F. C. McGough I. (2016). Atypical parkinsonism-associated retromer mutant alters endosomal sorting of specific cargo proteins. *J. Cell Biol.* 214 389–399. 10.1083/jcb.201604057 27528657 PMC4987296

[B147] McQuibban G. A. Lee J. R. Zheng L. Juusola M. Freeman M. (2006). Normal mitochondrial dynamics requires rhomboid-7 and affects *Drosophila* lifespan and neuronal function. *Curr. Biol.* 16 982–989. 10.1016/j.cub.2006.03.062 16713954

[B148] Melnattur K. V. Lee C.-H. (2011). Visual circuit assembly in *Drosophila*. *Dev. Neurobiol.* 71 1286–1296. 10.1002/dneu.20894 21538922 PMC4245071

[B149] Menzies F. M. Fleming A. Caricasole A. Bento C. F. Andrews S. P. Ashkenazi A. (2017). Autophagy and neurodegeneration: pathogenic mechanisms and therapeutic opportunities. *Neuron* 93 1015–1034. 10.1016/j.neuron.2017.01.022 28279350

[B150] Miklos G. L. Rubin G. M. (1996). The role of the genome project in determining gene function: insights from model organisms. *Cell* 86 521–529. 10.1016/S0092-8674(00)80126-9 8752207

[B151] Minke B. (2012). The history of the prolonged depolarizing afterpotential (PDA) and its role in genetic dissection of *Drosophila* phototransduction. *J. Neurogenet.* 26 106–117. 10.3109/01677063.2012.666299 22428622

[B152] Mizielinska S. Gronke S. Niccoli T. Ridler C. E. Clayton E. L. Devoy A. (2014). C9orf72 repeat expansions cause neurodegeneration in *Drosophila* through arginine-rich proteins. *Science* 345 1192–1194. 10.1126/science.1256800 25103406 PMC4944841

[B153] Morin C. Mitchell G. Larochelle J. Lambert M. Ogier H. Robinson B. H. (1993). Clinical, metabolic, and genetic aspects of cytochrome c oxidase deficiency in saguenay-lac-saint-jean. *Am. J. Hum. Genet.* 53 488–496. 8392291 PMC1682365

[B154] Morris A. A. Leonard J. V. Brown G. K. Bidouki S. K. Bindoff L. A. Woodward C. E. (1996). Deficiency of respiratory chain complex i is a common cause of leigh disease. *Ann. Neurol.* 40 25–30. 10.1002/ana.410400107 8687187

[B155] Mummery-Widmer J. L. Yamazaki M. Stoeger T. Novatchkova M. Bhalerao S. Chen D. (2009). Genome-wide analysis of notch signalling in *Drosophila* by transgenic RNAi. *Nature* 458 987–992. 10.1038/nature07936 19363474 PMC2988197

[B156] Naon D. Zaninello M. Giacomello M. Varanita T. Grespi F. Lakshminaranayan S. (2016). Critical reappraisal confirms that mitofusin 2 is an endoplasmic reticulum–mitochondria tether. *Proc. Natl. Acad. Sci. U.S.A.* 113 11249–11254. 10.1073/pnas.1606786113 27647893 PMC5056088

[B157] Neely G. G. Hess A. Costigan M. Keene A. C. Goulas S. Langeslag M. (2010). A genome-wide *Drosophila* screen for heat nociception identifies A2δ3 as an evolutionarily conserved pain gene. *Cell* 143 628–638. 10.1016/j.cell.2010.09.047 21074052 PMC3040441

[B158] Neukomm L. J. Burdett T. C. Gonzalez M. A. Züchner S. Freeman M. R. (2014). Rapid *in vivo* forward genetic approach for identifying axon death genes in *Drosophila*. *Proc. Natl. Acad. Sci. U.S.A.* 111 9965–9970. 10.1073/pnas.1406230111 24958874 PMC4103363

[B159] Newsome T. P. Asling B. Dickson B. J. (2000). Analysis of *Drosophila* photoreceptor axon guidance in eye-specific mosaics. *Development* 127 851–860. 10648243 10.1242/dev.127.4.851

[B160] Niccoli T. Partridge L. (2012). Ageing as a risk factor for disease. *Curr. Biol.* 22 R741–R752. 10.1016/J.CUB.2012.07.024 22975005

[B161] Niedzielska E. Smaga I. Gawlik M. Moniczewski A. Stankowicz P. Pera J. (2016). Oxidative stress in neurodegenerative diseases. *Mol. Neurobiol.* 53 4094–4125. 10.1007/s12035-015-9337-5 26198567 PMC4937091

[B162] Núñez M. T. Urrutia P. Mena N. Aguirre P. Tapia V. Salazar J. (2012). Iron toxicity in neurodegeneration. *Biometals* 25 761–776. 10.1007/s10534-012-9523-0 22318507

[B163] Nüsslein-Volhard C. (1994). Of flies and fishes. *Science* 266 572–574. 10.1126/science.7939708 7939708

[B164] O’Callaghan J. P. (2003). Neurotoxic esterase: not so toxic? *Nat. Genet.* 33 437–438. 10.1038/ng1135 12640456

[B165] Ohno M. Hiraoka Y. Matsuoka T. Tomimoto H. Takao K. Miyakawa T. (2009). Nardilysin regulates axonal maturation and myelination in the central and peripheral nervous system. *Nat. Neurosci.* 12 1506–1513. 10.1038/nn.2438 19935654

[B166] Ohsumi Y. (2014). Historical landmarks of autophagy research. *Cell Res.* 24 9–23. 10.1038/cr.2013.169 24366340 PMC3879711

[B167] Oláhová M. Yoon W. H. Thompson K. Jangam S. Fernandez L. Davidson J. M. (2018). Biallelic mutations in ATP5F1D, which encodes a subunit of ATP synthase, cause a metabolic disorder. *Am. J. Hum. Genet.* 102 494–504. 10.1016/j.ajhg.2018.01.020 29478781 PMC6117612

[B168] Oortveld M. A. W. Keerthikumar S. Oti M. Nijhof B. Fernandes A. C. Kochinke K. (2013). Human intellectual disability genes form conserved functional modules in *Drosophila*. *PLoS Genet.* 9:e1003911. 10.1371/journal.pgen.1003911 24204314 PMC3814316

[B169] Orem N. R. Xia L. Dolph P. J. (2006). An essential role for endocytosis of rhodopsin through interaction of visual arrestin with the AP-2 adaptor. *J. Cell Sci.* 119(Pt 15), 3141–3148. 10.1242/jcs.03052 16835270

[B170] O’Tousa J. E. (1992). Requirement of N-linked glycosylation site in *Drosophila* rhodopsin. *Vis. Neurosci.* 8 385–390. 10.1017/S0952523800004910 1534022

[B171] Pagliarini D. J. Calvo S. E. Chang B. Sheth S. A. Vafai S. B. Ong S.-E. (2008). A mitochondrial protein compendium elucidates complex i disease biology. *Cell* 134 112–123. 10.1016/j.cell.2008.06.016 18614015 PMC2778844

[B172] Park J. Al-Ramahi I. Tan Q. Mollema N. Diaz-Garcia J. R. Gallego-Flores T. (2013). RAS–MAPK–MSK1 pathway modulates ataxin 1 protein levels and toxicity in SCA1. *Nature* 498 325–331. 10.1038/nature12204 23719381 PMC4020154

[B173] Parks A. L. Cook K. R. Belvin M. Dompe N. A. Fawcett R. Huppert K. (2004). Systematic generation of high-resolution deletion coverage of the *Drosophila melanogaster* genome. *Nat. Genet.* 36 288–292. 10.1038/ng1312 14981519

[B174] Parzych K. R. Klionsky D. J. (2014). An overview of autophagy: morphology, mechanism, and regulation. *Antioxid. Redox Signal.* 20 460–473. 10.1089/ars.2013.5371 23725295 PMC3894687

[B175] Pellikka M. Tepass U. (2017). Unique cell biological profiles of retinal disease-causing missense mutations in the polarity protein crumbs. *J. Cell Sci.* 130 2147–2158. 10.1242/jcs.197178 28515229

[B176] Phillips S. E. Woodruff E. A. Liang P. Patten M. Broadie K. (2008). Neuronal loss of *Drosophila* NPC1a causes cholesterol aggregation and age-progressive neurodegeneration. *J. Neurosci.* 28 6569–6582. 10.1523/JNEUROSCI.5529-07.2008 18579730 PMC3306184

[B177] Pickard G. E. Sollars P. J. (2011). Intrinsically photosensitive retinal ganglion cells. *Rev. Physiol. Biochem. Pharmacol.* 162 59–90. 10.1007/112_2011_4 22160822

[B178] Quintana A. Kruse S. E. Kapur R. P. Sanz E. Palmiter R. D. (2010). Complex I deficiency due to loss of Ndufs4 in the brain results in progressive encephalopathy resembling leigh syndrome. *Proc. Natl. Acad. Sci. U.S.A.* 107 10996–11001. 10.1073/pnas.1006214107 20534480 PMC2890717

[B179] Quintana A. Morgan P. G. Kruse S. E. Palmiter R. D. Sedensky M. M. (2012). Altered anesthetic sensitivity of mice lacking Ndufs4, a subunit of mitochondrial complex I. *PLoS One* 7:e42904. 10.1371/journal.pone.0042904 22912761 PMC3422219

[B180] Rahman M. Ham H. Liu X. Sugiura Y. Orth K. Krämer H. (2012). Visual neurotransmission in *Drosophila* requires expression of fic in glial capitate projections. *Nat. Neurosci.* 15 871–875. 10.1038/nn.3102 22544313 PMC3578554

[B181] Rainier S. Bui M. Mark E. Thomas D. Tokarz D. Ming L. (2008). Neuropathy target esterase gene mutations cause motor neuron disease. *Am. J. Hum. Genet.* 82 780–785. 10.1016/j.ajhg.2007.12.018 18313024 PMC2427280

[B182] Ravikumar B. Vacher C. Berger Z. Davies J. E. Luo S. Oroz L. G. (2004). Inhibition of MTOR induces autophagy and reduces toxicity of polyglutamine expansions in fly and mouse models of huntington disease. *Nat. Genet.* 36 585–595. 10.1038/ng1362 15146184

[B183] Reuter J. E. Nardine T. M. Penton A. Billuart P. Scott E. K. Usui T. (2003). A mosaic genetic screen for genes necessary for *Drosophila* mushroom body neuronal morphogenesis. *Development* 130 1203–1213. 10.1242/dev.00319 12571111

[B184] Rivera-Alba M. Vitaladevuni S. N. Mishchenko Y. Lu Z. Takemura S. Scheffer L. (2011). Wiring economy and volume exclusion determine neuronal placement in the drosophila brain. *Curr. Biol.* 21 2000–2005. 10.1016/j.cub.2011.10.022 22119527 PMC3244492

[B185] Robak L. A. Jansen I. E. Rooij J. van Uitterlinden A. G. Kraaij R. Jankovic J. (2017). Excessive burden of lysosomal storage disorder gene variants in parkinson’s disease. *Brain* 140 3191–3203. 10.1093/brain/awx285 29140481 PMC5841393

[B186] Roy B. Jackson G. R. (2014). Interactions between tau and α-synuclein augment neurotoxicity in a *Drosophila* model of parkinson’s disease. *Hum. Mol. Genet.* 23 3008–3023. 10.1093/hmg/ddu011 24430504 PMC4014195

[B187] Ryder E. Blows F. Ashburner M. Bautista-Llacer R. Coulson D. Drummond J. (2004). The drosdel collection: a set of P-element insertions for generating custom chromosomal aberrations in *Drosophila Melanogaster*. *Genetics* 167 797–813. 10.1534/genetics.104.026658 15238529 PMC1470913

[B188] Şahin A. Held A. Bredvik K. Major P. Achilli T.-M. Kerson A. G. (2017). Human SOD1 ALS mutations in a *Drosophila* knock-in model cause severe phenotypes and reveal dosage-sensitive gain- and loss-of-function components. *Genetics* 205 707–723. 10.1534/genetics.116.190850 27974499 PMC5289846

[B189] Saj A. Arziman Z. Stempfle D. Belle W. van Sauder U. Horn T. (2010). A combined *ex vivo* and *in vivo* rnai screen for notch regulators in drosophila reveals an extensive notch interaction network. *Dev. Cell* 18 862–876. 10.1016/j.devcel.2010.03.013 20493818

[B190] Salazar J. L. Yamamoto S. (2018). Integration of *Drosophila* and human genetics to understand notch signaling related diseases. *Adv. Exp. Med. Biol.* 1066 141–185. 10.1007/978-3-319-89512-3_8 30030826 PMC6233323

[B191] Sando S. B. Melquist S. Cannon A. Hutton M. L. Sletvold O. Saltvedt I. (2008). APOE E4 lowers age at onset and is a high risk factor for alzheimer’s disease; a case control study from central norway. *BMC Neurol.* 8:9. 10.1186/1471-2377-8-9 18416843 PMC2375917

[B192] Sandoval H. Yao C.-K. Chen K. Jaiswal M. Donti T. Lin Y. Q. (2014). Mitochondrial fusion but not fission regulates larval growth and synaptic development through steroid hormone production. *eLife* 3:e03558. 10.7554/eLife.03558 25313867 PMC4215535

[B193] Satoh A. K. O’Tousa J. E. Ozaki K. Ready D. F. (2005). Rab11 mediates post-golgi trafficking of rhodopsin to the photosensitive apical membrane of *Drosophila* photoreceptors. *Development* 132 1487–1497. 10.1242/dev.01704 15728675

[B194] Satoh A. K. Ready D. F. (2005). Arrestin1 mediates light-dependent rhodopsin endocytosis and cell survival. *Curr. Biol.* 15 1722–1733. 10.1016/j.cub.2005.08.064 16213818

[B195] Satoh T. Ohba A. Liu Z. Inagaki T. Satoh A. K. (2015). DPob/EMC is essential for biosynthesis of rhodopsin and other multi-pass membrane proteins in *Drosophila* photoreceptors. *eLife* 4:e06306. 10.7554/eLife.06306 25715730 PMC4341237

[B196] Saura C. A. Choi S.-Y. Beglopoulos V. Malkani S. Zhang D. Shankaranarayana Rao B. S. (2004). Loss of presenilin function causes impairments of memory and synaptic plasticity followed by age-dependent neurodegeneration. *Neuron* 42 23–36. 10.1016/S0896-6273(04)00182-5 15066262

[B197] Sega G. A. (1984). A review of the genetic effects of ethyl methanesulfonate. *Mutat. Res.* 134 113–142. 6390190 10.1016/0165-1110(84)90007-1

[B198] Şentürk M. Bellen H. J. (2018). Genetic strategies to tackle neurological diseases in fruit flies. *Curr. Opin. Neurobiol.* 50 24–32. 10.1016/j.conb.2017.10.017 29128849 PMC5940587

[B199] Shiraishi R. Tamura T. Sone M. Okazawa H. (2014). Systematic analysis of fly models with multiple drivers reveals different effects of ataxin-1 and huntingtin in neuron subtype-specific expression. *PLoS One* 9:e116567. 10.1371/journal.pone.0116567 25551764 PMC4281079

[B200] Slack C. Somers W. Sousa-Nunes R. Chia W. Overton P. M. (2006). A mosaic genetic screen for novel mutations affecting *Drosophila* neuroblast divisions. *BMC Genet.* 7:33. 10.1186/1471-2156-7-33 16749923 PMC1523195

[B201] Sobreira N. Schiettecatte F. Valle D. Hamosh A. (2015). GeneMatcher: a matching tool for connecting investigators with an interest in the same gene. *Hum. Mutat.* 36 928–930. 10.1002/humu.22844 26220891 PMC4833888

[B202] St Johnston D. (2002). The art and design of genetic screens: *Drosophila melanogaster*. *Nat. Rev. Genet.* 3 176–188. 10.1038/nrg751 11972155

[B203] Stephenson E. Nathoo N. Mahjoub Y. Dunn J. F. Yong V. W. (2014). Iron in multiple sclerosis: roles in neurodegeneration and repair. *Nat. Rev. Neurol.* 10 459–468. 10.1038/nrneurol.2014.118 25002107

[B204] Sterky F. H. Ruzzenente B. Gustafsson C. M. Samuelsson T. Larsson N.-G. (2010). LRPPRC is a mitochondrial matrix protein that is conserved in metazoans. *Biochem. Biophys. Res. Commun.* 398 759–764. 10.1016/J.BBRC.2010.07.019 20633537

[B205] Stowers R. S. Schwarz T. L. (1999). A genetic method for generating drosophila eyes composed exclusively of mitotic clones of a single genotype. *Genetics* 152 1631–1639. 10430588 10.1093/genetics/152.4.1631 PMC1460682

[B206] Tabuchi K. Chen G. Sudhof T. C. Shen J. (2009). Conditional forebrain inactivation of nicastrin causes progressive memory impairment and age-related neurodegeneration. *J. Neurosci.* 29 7290–7301. 10.1523/JNEUROSCI.1320-09.2009 19494151 PMC2719251

[B207] Tan K. L. Haelterman N. A. Kwartler C. S. Regalado E. S. Lee P.-T. Nagarkar-Jaiswal S. (2018). Ari-1 regulates myonuclear organization together with parkin and is associated with aortic aneurysms. *Dev. Cell* 45 226.e8–244.e8. 10.1016/j.devcel.2018.03.020 29689197 PMC5920516

[B208] Tang G. Gudsnuk K. Kuo S.-H. Cotrina M. L. Rosoklija G. Sosunov A. (2014). Loss of MTOR-dependent macroautophagy causes autistic-like synaptic pruning deficits. *Neuron* 83 1131–1143. 10.1016/j.neuron.2014.07.040 25155956 PMC4159743

[B209] Thiffault I. Rioux M. F. Tetreault M. Jarry J. Loiselle L. Poirier J. (2006). A new autosomal recessive spastic ataxia associated with frequent white matter changes maps to 2q33-34. *Brain* 129 2332–2340. 10.1093/brain/awl110 16672289

[B210] Tian X. Gala U. Zhang Y. Shang W. Nagarkar Jaiswal S. di Ronza A. (2015). A voltage-gated calcium channel regulates lysosomal fusion with endosomes and autophagosomes and is required for neuronal homeostasis. *PLoS Biol.* 13:e1002103. 10.1371/journal.pbio.1002103 25811491 PMC4374850

[B211] Tottene A. Fellin T. Pagnutti S. Luvisetto S. Striessnig J. Fletcher C. (2002). Familial hemiplegic migraine mutations increase Ca2+ influx through single human CaV2.1 channels and decrease maximal CaV2.1 current density in neurons. *Proc. Natl. Acad. Sci. U.S.A.* 99 13284–13289. 10.1073/pnas.192242399 12235360 PMC130625

[B212] Ugur B. Bao H. Stawarski M. Duraine L. R. Zuo Z. Lin Y. Q. (2017). The krebs cycle enzyme isocitrate dehydrogenase 3A couples mitochondrial metabolism to synaptic transmission. *Cell Rep.* 21 3794–3806. 10.1016/j.celrep.2017.12.005 29281828 PMC5747319

[B213] Van Den Brink D. M. Cubizolle A. Chatelain G. Davoust N. Girard V. Johansen S. (2018). Physiological and pathological roles of FATP-mediated lipid droplets in *Drosophila* and mice retina. *PLoS Genet.* 14:e1007627. 10.1371/journal.pgen.1007627 30199545 PMC6147681

[B214] Végh M. Basler K. (2003). A genetic screen for hedgehog targets involved in the maintenance of the *Drosophila* anteroposterior compartment boundary. *Genetics* 163 1427–1438. 12702686 10.1093/genetics/163.4.1427 PMC1462513

[B215] Venken K. J. T. Carlson J. W. Schulze K. L. Pan H. He Y. Spokony R. (2009). Versatile P[Acman] BAC libraries for transgenesis studies in *Drosophila melanogaster*. *Nat. Methods* 6 431–434. 10.1038/nmeth.1331 19465919 PMC2784134

[B216] Venken K. J. T. He Y. Hoskins R. A. Bellen H. J. (2006). P[Acman]: ABAC transgenic platform for targeted insertion of large DNA fragments in *D. melanogaster.* *Science* 314 1747–1751. 10.1126/science.1134426 17138868

[B217] Venken K. J. T. Popodi E. Holtzman S. L. Schulze K. L. Park S. Carlson J. W. (2010). A molecularly defined duplication set for the x chromosome of *Drosophila melanogaster*. *Genetics* 186 1111–1125. 10.1534/genetics.110.121285 20876565 PMC2998297

[B218] Verstreken P. Koh T.-W. Schulze K. L. Zhai R. G. Hiesinger P. R. Zhou Y. (2003). Synaptojanin Is recruited by endophilin to promote synaptic vesicle uncoating. *Neuron* 40 733–748. 10.1016/S0896-6273(03)00644-5 14622578

[B219] Verstreken P. Ly C. V. Venken K. J. T. Koh T.-W. Zhou Y. Bellen H. J. (2005). Synaptic mitochondria are critical for mobilization of reserve pool vesicles at *Drosophila* neuromuscular junctions. *Neuron* 47 365–378. 10.1016/j.neuron.2005.06.018 16055061

[B220] Verstreken P. Ohyama T. Haueter C. Habets R. L. P. Lin Y. Q. Swan L. E. (2009). Tweek, an evolutionarily conserved protein, is required for synaptic vesicle recycling. *Neuron* 63 203–215. 10.1016/j.neuron.2009.06.017 19640479 PMC2759194

[B221] Vilariño-Güell C. Wider C. Ross O. A. Dachsel J. C. Kachergus J. M. Lincoln S. J. (2011). VPS35 mutations in parkinson disease. *Am. J. Hum. Genet.* 89 162–167. 10.1016/j.ajhg.2011.06.001 21763482 PMC3135796

[B222] Walsh E. P. Brown N. H. (1998). A screen to identify drosophila genes required for integrin-mediated adhesion. *Genetics* 150 791–805. 9755209 10.1093/genetics/150.2.791 PMC1460349

[B223] Wang L. Schuster G. U. Hultenby K. Zhang Q. Andersson S. Gustafsson J.-A. (2002). Liver X receptors in the central nervous system: from lipid homeostasis to neuronal degeneration. *Proc. Natl. Acad. Sci. U.S.A.* 99 13878–13883. 10.1073/pnas.172510899 12368482 PMC129791

[B224] Wang S. Tan K. L. Agosto M. A. Xiong B. Yamamoto S. Sandoval H. (2014). The retromer complex is required for rhodopsin recycling and its loss leads to photoreceptor degeneration. *PLoS Biol.* 12:e1001847. 10.1371/journal.pbio.1001847 24781186 PMC4004542

[B225] Wang T. Montell C. (2007). Phototransduction and retinal degeneration in *Drosophila*. *Pflügers Arch. Eur. J. Physiol.* 454 821–847. 10.1007/s00424-007-0251-1 17487503

[B226] Wangler M. F. Yamamoto S. Bellen H. J. (2015). Fruit flies in biomedical research. *Genetics* 199 639–653. 10.1534/genetics.114.171785 25624315 PMC4349060

[B227] Wangler M. F. Yamamoto S. Chao H.-T. Posey J. E. Westerfield M. Postlethwait J. (2017). Model organisms facilitate rare disease diagnosis and therapeutic research. *Genetics* 207 9–27. 10.1534/genetics.117.203067 28874452 PMC5586389

[B228] Wappl E. Koschak A. Poteser M. Sinnegger M. J. Walter D. Eberhart A. (2002). Functional consequences of P/Q-type Ca 2+ channel ca v 2.1 missense mutations associated with episodic ataxia type 2 and progressive ataxia. *J. Biol. Chem.* 277 6960–6966. 10.1074/jbc.M110948200 11742003

[B229] Waterham H. R. Koster J. Roermund C. W. T. van Mooyer P. A. W. Wanders R. J. A. Leonard J. V. (2007). A lethal defect of mitochondrial and peroxisomal fission. *N. Engl. J. Med.* 356 1736–1741. 10.1056/NEJMoa064436 17460227

[B230] Welte M. A. (2015). Expanding roles for lipid droplets. *Curr. Biol.* 25 R470–R481. 10.1016/j.cub.2015.04.004 26035793 PMC4452895

[B231] Westermann B. (2010). Mitochondrial dynamics in model organisms: what yeasts, worms and flies have taught us about fusion and fission of mitochondria. *Semin. Cell Dev. Biol.* 21 542–549. 10.1016/j.semcdb.2009.12.003 20006727

[B232] Weuve J. Hebert L. E. Scherr P. A. Evans D. A. (2014). Deaths in the United States among persons with Alzheimer’s Disease (2010–2050). *Alzheimers Dement.* 10 e40–e46. 10.1016/j.jalz.2014.01.004 24698031 PMC3976898

[B233] Wieschaus E. Nüsslein-Volhard C. Jürgens G. (1984). Mutations affecting the pattern of the larval cuticle in *Drosophila melanogaster*. *Wilhelm Rouxs Arch. Dev. Biol.* 193 296–307. 10.1007/BF00848158 28305339

[B234] Winkler S. Schwabedissen A. Backasch D. Bökel C. Seidel C. Bönisch S. (2005). Target-selected mutant screen by tilling in *Drosophila.* *Genome Res.* 15 718–723. 10.1101/gr.3721805 15867432 PMC1088300

[B235] Winrow C. J. Hemming M. L. Allen D. M. Quistad G. B. Casida J. E. Barlow C. (2003). Loss of neuropathy target esterase in mice links organophosphate exposure to hyperactivity. *Nat. Genet.* 33 477–485. 10.1038/ng1131 12640454

[B236] Wu M. Gu J. Guo R. Huang Y. Yang M. (2016). Structure of mammalian respiratory supercomplex I1III2IV1. *Cell* 167 1598.e10–1609.e10. 10.1016/J.CELL.2016.11.012 27912063

[B237] Xiong B. Bayat V. Jaiswal M. Zhang K. Sandoval H. Charng W.-L. (2012). Crag Is a GEF for Rab11 required for rhodopsin trafficking and maintenance of adult photoreceptor cells. *PLoS Biol.* 10:e1001438. 10.1371/journal.pbio.1001438 23226104 PMC3514319

[B238] Xiong B. Bellen H. J. (2013). Rhodopsin homeostasis and retinal degeneration: lessons from the fly. *Trends Neurosci.* 36 652–660. 10.1016/j.tins.2013.08.003 24012059 PMC3955215

[B239] Xiong Y. Yu J. (2018). Modeling parkinson’s disease in drosophila: what have we learned for dominant traits? *Front. Neurol.* 9:228 10.3389/fneur.2018.00228 PMC5900015 29686647

[B240] Xu W. Barrientos T. Andrews N. C. (2013). Iron and copper in mitochondrial diseases. *Cell Metab.* 17 319–328. 10.1016/J.CMET.2013.02.004 23473029 PMC3594794

[B241] Yamamoto S. Bayat V. Bellen H. J. Tan C. (2013). Protein Phosphatase 1ß limits ring canal constriction during *Drosophila* germline cyst formation. *PLoS One* 8:e70502. 10.1371/journal.pone.0070502 23936219 PMC3723691

[B242] Yamamoto S. Charng W.-L. Rana N. A. Kakuda S. Jaiswal M. Bayat V. (2012). A mutation in EGF repeat-8 of notch discriminates between serrate/jagged and delta family ligands. *Science* 338 1229–1232. 10.1126/science.1228745 23197537 PMC3663443

[B243] Yamamoto S. Jaiswal M. Charng W.-L. Gambin T. Karaca E. Mirzaa G. (2014). A *Drosophila* genetic resource of mutants to study mechanisms underlying human genetic diseases. *Cell* 159 200–214. 10.1016/j.cell.2014.09.002 25259927 PMC4298142

[B244] Yarosh W. Monserrate J. Tong J. J. Tse S. Le P. K. Nguyen K. (2008). The molecular mechanisms of opa1-mediated optic atrophy in *Drosophila* model and prospects for antioxidant treatment. *PLoS Genet.* 4:e6. 10.1371/journal.pgen.0040006 18193945 PMC2174975

[B245] Yoon W. H. Sandoval H. Nagarkar-Jaiswal S. Jaiswal M. Yamamoto S. Haelterman N. A. (2017). Loss of nardilysin, a mitochondrial co-chaperone for α-ketoglutarate dehydrogenase, promotes MTORC1 activation and neurodegeneration. *Neuron* 93 115–131. 10.1016/j.neuron.2016.11.038 28017472 PMC5242142

[B246] Yu J.-T. Tan L. Hardy J. (2014). Apolipoprotein E in alzheimer’s disease: an update. *Annu. Rev. Neurosci.* 37 79–100. 10.1146/annurev-neuro-071013-014300 24821312

[B247] Zhai R. G. Cao Y. Hiesinger P. R. Zhou Y. Mehta S. Q. Schulze K. L. (2006). Drosophila NMNAT maintains neural integrity independent of its NAD synthesis activity. *PLoS Biol.* 4:e416. 10.1371/journal.pbio.0040416 17132048 PMC1665629

[B248] Zhang K. Li Z. Jaiswal M. Bayat V. Xiong B. Sandoval H. (2013). The C8ORF38 homologue sicily is a cytosolic chaperone for a mitochondrial complex I subunit. *J. Cell Biol.* 200 807–820. 10.1083/jcb.201208033 23509070 PMC3601355

[B249] Zhang X. (2014). Exome sequencing greatly expedites the progressive research of mendelian diseases. *Front. Med.* 8 42–57. 10.1007/s11684-014-0303-9 24384736

[B250] Zhao X.-L. Wang W.-A. Tan J.-X. Huang J.-K. Zhang X. Zhang B.-Z. (2010). Expression of beta-amyloid induced age-dependent presynaptic and axonal changes in *Drosophila*. *J. Neurosci.* 30 1512–1522. 10.1523/JNEUROSCI.3699-09.2010 20107079 PMC6633795

[B251] Zhuchenko O. Bailey J. Bonnen P. Ashizawa T. Stockton D. W. Amos C. (1997). Autosomal dominant cerebellar ataxia (SCA6) associated with small polyglutamine expansions in the α1a-voltage-dependent calcium channel. *Nat. Genet.* 15 62–69. 10.1038/ng0197-62 8988170

[B252] Zimprich A. Benet-Pagès A. Struhal W. Graf E. Eck S. H. Offman M. N. (2011). A mutation in VPS35, encoding a subunit of the retromer complex, causes late-onset parkinson disease. *Am. J. Hum. Genet.* 89 168–175. 10.1016/j.ajhg.2011.06.008 21763483 PMC3135812

[B253] Züchner S. Mersiyanova I. V. Muglia M. Bissar-Tadmouri N. Rochelle J. Dadali E. L. (2004). Mutations in the mitochondrial GTPase mitofusin 2 cause charcot-marie-tooth neuropathy type 2A. *Nat. Genet.* 36 449–451. 10.1038/ng1341 15064763

